# Perceptions of environmental changes among a climate-vulnerable population from Bangladesh

**DOI:** 10.1007/s10584-024-03678-6

**Published:** 2024-02-01

**Authors:** Jan Freihardt

**Affiliations:** Center for Comparative and International Studies (CIS), ETH Zurich, 8092 Zurich, Switzerland

**Keywords:** Environmental perceptions, Riverbank erosion, Climate change literacy, Survey data, Satellite imagery, Bangladesh

## Abstract

Effective climate change adaptation requires a thorough understanding of whether and how affected populations perceive climatic and environmental changes. Existing research has been inconclusive regarding the consistency of these perceptions compared to objective meteorological indicators. Moreover, no systematic comparison has been done for the perception of discrete environmental events such as floods or erosion. This study relies on novel panel survey data of approximately 1700 households residing along the Jamuna River in Bangladesh as well as on unique individual-level, satellite-based erosion data. It compares respondents’ perceptions of environmental events, namely riverbank erosion, and three climate change indicators, specifically long-term temperature change and changes in precipitation during wet and dry seasons, to objective measurements using satellite imagery and climatic time-series data (CRU TS). I find that long-term temperature change is perceived more accurately than long-term changes in precipitation. Given that educational attainment and climate change literacy among the study population are low, this indicates that global temperature increases are felt even by remote populations who have never heard the term climate change. Erosion is strongly overestimated, especially by those respondents who had been personally affected by it. Since human behavior is guided by perceptions rather than objective data, this has important policy implications, underlining the importance of considering people’s perceptions if the goal is to assist them in adapting to environmental changes.

**Supplementary Information:**

The online version contains supplementary material available at 10.1007/s10584-024-03678-6.

## Introduction

Anthropogenic climate change affects the intensity, duration, and frequency of many atmospheric, hydrologic, geologic, and biologic hazards (IPCC 2018, 2021; Stott et al. 2016). Affected populations are faced with the challenge of adapting to unprecedented frequencies and intensities of environmental conditions and events. Depending on the context, different adaptation options arise. In the agricultural sector, farmers who perceive the climate to change have been found to engage in various adaptation strategies, ranging from in-situ adaptation, e.g., planting drought-resistant crops and implementing additional irrigation practices, to migration to other locations with more favorable environmental conditions (Al-Amin et al. 2019; de Longueville et al. 2020; Elum et al. 2017; Hasan and Kumar 2019). For climate change adaptation to be effective, it is crucial to understand whether and how affected populations perceive environmental changes (Dessai et al. 2004).

Understanding environmental perceptions^1^ is of particular relevance for countries of the Majority World, which are often characterized by a large population share whose livelihoods are directly dependent on the environment (e.g., through farming or fishing). Yet, despite a wealth of studies comparing environmental perceptions with meteorological evidence, previous research has been inconclusive with respect to the accuracy^2^ of these perceptions. While some studies show that actual weather data and reported data converge (Alam et al. 2017 and Hasan and Kumar 2019 (Bangladesh); Kosmowski et al. 2016 (Niger); Shrestha et al. 2019 (Nepal); Linke et al. 2020 (Kenya)), others show that they converge only among certain populations (Koubi et al. 2016 (Vietnam)), or diverge completely (Meze-Hausken 2004 (Ethiopia); Moyo et al. 2012 (Zimbabwe); Sutcliffe et al. 2016 (Malawi)).

This paper contributes to the literature by presenting evidence on the relationship between objective data and individuals’ perceptions of environmental and climatic changes^3^ from a pre-registered survey among a rural population in Bangladesh (*N* = 1698). Specifically, it examines how accurately three different environmental and climatic indicators are perceived: rainfall, temperature, and riverbank erosion. Note that these comprise both gradual (temperature, precipitation) and sudden-onset environmental changes (erosion).

This distinction is important, given that the accuracy of perceptions depends on the environmental parameter considered: The extant literature agrees that perceptions about temperature are more consistent with meteorological evidence than perceptions about rainfall (de Longueville et al. 2020; Abid et al. 2019; Madhuri and Sharma, 2020; Osbahr et al. 2011; though see Marlon et al. 2019). However, a systematic comparison between objective data and subjective perceptions has not yet been conducted in the realm of discrete environmental events such as floods, storms, or riverbank erosion (Howe et al. 2019). While several studies investigate perceptions of riverbank erosion (Alam et al. 2017; Das 2011; Hasan and Kumar 2019), none of them compares these individual perceptions to objectively measured data.

Riverbank erosion—meaning that bank material gets carried away by the flow of water—can occur either gradually in small amounts (fluvial erosion) or abruptly in large quantities (mass failure). Especially, the latter is of high societal relevance, given that mass failure events can erode agricultural land and/or destroy houses or infrastructure, such as roads within a short time. In Bangladesh alone, riverbank erosion affects several hundred thousand people each year. Yet, there is no empirical evidence on how affected populations perceive such events. Since rapid-onset events have different properties than gradual changes (e.g., in temperature or precipitation), the results from the large body of literature on temperature and precipitation perceptions might have limited applicability for rapid-onset events. Studying the accuracy of erosion perceptions hence makes a significant contribution to the literature.^4^

In addition, this paper investigates which factors influence the accuracy of environmental perceptions. Accurate perceptions can lead to improved adaptation compared to biased perceptions, which are more likely to lead to maladaptation (Abid et al. 2019). For instance, Sutcliffe et al. (2016) found that Malawian farmers increasingly opt for short-season maize varieties based on a perception of decreasing season lengths due to climate change. These perceptions are, however, not in line with meteorological data, meaning that farmers might achieve lower yields than when cultivating longer-maturing varieties, hence reducing their capital and resilience towards longer-term climate change. Thus, identifying which segments of the population are particularly prone to under- or overestimate environmental changes is of concern for policymakers who wish to support populations in adapting effectively to climatic changes.

Theoretically, I argue that the accuracy of environmental perceptions is influenced by the underlying psychological processing. I expect local extreme events such as riverbank erosion to be perceived inaccurately, as opposed to long-term climatic changes such as temperature or precipitation. Empirically, I compare novel survey data of around 1700 household heads residing along the 250 km of the Jamuna River in Bangladesh to objective measurements using satellite imagery and climatic time-series data (CRU TS). I find that long-term temperature changes are perceived more in line with meteorological evidence than changes in precipitation, confirming previous literature findings. Riverbank erosion is strongly overestimated, both in absolute terms and in time trends, especially by those respondents who had been personally affected by it. Environmentally dependent respondents perceive environmental changes less accurately than those whose income does not depend on the environment, and this holds when controlling for education. Long-term climatic changes are felt even by respondents with low educational attainment and who have never heard the term climate change, suggesting that climate change perceptions are not solely driven by climate literacy. Overall, this study is the first to present evidence of misperceptions of discrete environmental events among a population which is highly vulnerable to climatic and environmental changes.

## Perceptions of environmental change

While there is a considerable body of empirical evidence about the accuracy of subjective environmental perceptions with regard to objective meteorological data (see Madhuri and Sharma, 2020 for a review), the theoretical underpinnings of these findings are not always made explicit. Those studies that derive theoretical explanations typically employ one of two psychological theories.

First, the dual-process theory, as outlined by Kahneman (2011), claims that the human brain can process information either experientially (System 1) or analytically (System 2). While experiential processing is fast and driven by affect, analytical processing is more conscious, abstract, and slow. Although it is oftentimes assumed that people process climate information (e.g., stemming from risk communication) analytically, people also rely heavily on experiential processing (Marx et al. 2007). Experiential processing might, however, be less accurate than analytical processing, given that it is fast and that accuracy has been found to decrease under time pressure (Fraser-Mackenzie and Dror 2011). Second, the construal-level theory argues that the “psychological distance” of different stimuli influences how we process them (Trope and Liberman 2010): The more abstract and distant a stimulus is, the more it will be processed analytically, whereas close stimuli are more likely to be processed experientially. Distance herein refers not only to space but likewise to time and hypotheticality.

Howe et al. (2019) relate these two concepts to the processing of weather and climate information. More locally and directly perceived experiences like the ambient temperature are more likely to be processed experientially (see also Zaval et al. 2014) than global or long-term trends such as a 20-year temperature increase. Local extreme events take an intermediate position between experiential and analytical processing because they are less directly perceived than, for example, the ambient temperature but also less abstract than, for example, a long-term temperature trend (Howe et al. 2019).

**Fig. 1:**
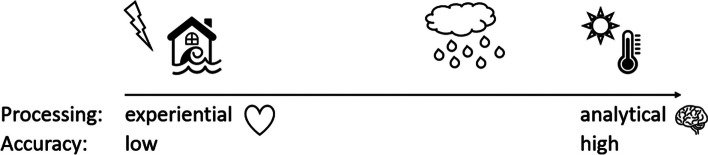
Theoretical expectations for the accuracy of perceptions of riverbank erosion, precipitation, and temperature

Relating these theoretical concepts to the first research question about the accuracy of perceiving different environmental changes, I expect local, sudden-onset environmental change (in this case, riverbank erosion) to be processed experientially and, hence, not accurately with respect to an objective baseline (Fig. 1). In contrast, long-term, gradual environmental change (in this case: long-term changes in temperature and precipitation) should be processed analytically and, thus, accurately in comparison to meteorological evidence.

- ***H1***: *Local, sudden-onset environmental events (i.e., riverbank erosion) are not perceived accurately compared to objective data.*
- ***H2***: *Long-term, gradual environmental changes (i.e., temperature and precipitation) are perceived accurately compared to meteorological data.*

Hypothesis 2 implies that from the perspective of psychological distance, there should be no difference in perceptions of long-term changes in temperature and precipitation since both can be categorized as gradual trends. Still, the two climatic indicators have different characteristics: While temperature can be felt every day, precipitation occurs on some days, while it does not occur on other days. Likewise, many regions of the world experience a distinct rainy season followed by a dry season. This discrete nature of precipitation might make it harder to estimate long-term trends accurately since perceptions are more prone to be biased by particular rainfall events^5^:

- ***H3***: *Long-term changes in temperature are perceived more accurately than long-term changes in precipitation.*

The accuracy of environmental perceptions also depends on different mediating factors. First, people with environmentally dependent occupations are more sensitive to climatic and environmental changes and can thus be expected to pay close attention to environmental changes. Accordingly, several studies show that climate-sensitive households have a higher level of accuracy than non-sensitive ones (Kosmowski et al. 2016; Shrestha et al. 2019). By contrast, Linke et al. (2020) do not find differences in perceptions between respondents with or without agricultural jobs. Second, the longer someone lives in a place, the more likely she is to perceive long-term changes accurately, in contrast to newly arrived residents. This effect will be stronger the more different the new location of the newly arrived residents is compared to the region of their origin. Empirical evidence confirms this expectation (Shrestha et al. 2019). Third, given that I argue that erosion events are processed experientially rather than analytically, their perception might be related to affect when recalling a specific event. Affect, in turn, is strongly influenced by personally experiencing severe impacts of a natural disaster (Siegrist and Gutscher 2008).^6^ Similarly, Osbahr et al. (2011) argue that the impact on livelihoods shapes peoples’ perception of environmental changes. Therefore, I expect respondents who were personally affected by past erosion events to perceive these less accurately than unaffected ones. Lastly, in line with the concept of “psychological distance” introduced above, I expect respondents who live closer to the riverbank to perceive erosion more accurately than those who live further away.

With respect to the perception of climate change-related indicators (i.e., temperature and precipitation), I argue that climate change literacy can have a priming effect on perceptions related to underlying psychological mechanisms such as motivated reasoning (Kunda 1990) or confirmation bias (Nickerson 1998). In other words, if someone *knows* that climate change exists, she is more likely to state that she also *feels* it. Indeed, it has been found that beliefs about global warming can bias perceptions of local climate conditions (Howe 2018; Howe and Leiserowitz 2013). Hence, I expect respondents who are familiar with the concept of climate change to show stronger perceptions of temperature increases and precipitation decreases than those who are unfamiliar with climate change.

## Research design

### The case: Jamuna River in Bangladesh

Bangladesh is among the countries most susceptible to the adverse effects of climate change, due to its topography and its location in one of the largest river deltas of the world (Rigaud et al. 2018). It is affected heavily by sea level rise, frequent cyclones, and high monsoon rainfall that increases river flow, which in turn contributes to extensive flooding and riverbank erosion (Hasan et al. 2018; Islam et al. 2021). In Bangladesh, riverbank/coastal erosion is among the most impactful processes in terms of yearly economic damage (Ahmed 2015).

Around 20 out of 64 districts in the country are prone to riverbank erosion, which consumes around 8700 ha of land each year (Alam 2017) and thereby affects more than 500.000 people (Kaiser 2023). While communities along the rivers are aware of erosion risks, people choose to settle next to rivers due to the high soil fertility and/or lack of other suitable space given the country’s high population density. Erosion has several negative impacts on affected communities, including the destruction of farmable land, housing, and infrastructure such as roads, schools, and hospitals. Along the 250 km long Jamuna River, the case region of this study, net erosion was about 933 km^2^ during the 1973–2017 period (CEGIS 2018). This would correspond to a widening of the riverbank of more than 4 km if the erosion was distributed evenly along the length of the river. In certain areas, erosion causes an inland shift of the riverbank by several hundred meters per year. Erosion events occur mainly during the rainy monsoon season, typically from June to October. Since the river erodes more land than it can transport, a part of the eroded land is deposited downstream and forms new land in the form of islands (so-called *chars*). However, the river erodes approximately seven times more land than it forms (Sarker et al. 2014).

The study region exhibits a warm, humid climate characterized by a monsoon season from June to August, a dry season from November to March, and transition seasons in between (see climate diagram in Fig. S3).

### Survey overview

For the empirical analysis, I use cross-sectional data from the first wave of a panel survey among 1698 household heads from 36 locations distributed along the whole length of the Jamuna River in Bangladesh (see map in Fig. S1), conducted in June and July 2021. Participants were selected in a multi-stage cluster design (see Appendix A for details on the selection procedure). In the first stage, all survey locations (1-km stretches) potentially at risk of riverbank erosion along the easternmost riverbank line of the Jamuna River were identified. Of all potential locations, 36 (86%) could be visited. At each of these 36 locations, households were sampled using a stratified random spatial sampling design to survey households located within three zones defined by distance from the shoreline. Hence, I am confident that the respondents constitute a high-quality sample^7^ of the riverbank population at risk of erosion in Bangladesh. The respondents are 87% male, on average 48 years old, and are mostly illiterate or have only primary education (Table S2). 56% of the respondents depend on the environment as their primary income source, either by working on their own or others’ agricultural land. Other common income sources are owning a small business/shop, non-agriculture-related day labor, remittances, transport, and textile weaving. Interviews were conducted face-to-face in Bangla by native interviewers using Qualtrics and lasted for about 45–60 min. The questionnaire included both closed and open-ended questions pertaining to respondents’ experience with environmental events as well as personal and household information. The study has been pre-registered at OSF; details are provided in Appendix D.

### Erosion and climate data

To assess the objective erosion extent at the riverbank closest to the respondents’ houses, I extracted the easternmost shoreline of the Jamuna in January of the years 2015 to 2021. These shorelines were drawn manually in the Google Earth Engine, using optical Sentinel-2 satellite images and the erosion assessment algorithm developed by Freihardt and Frey (2023). Using the households’ coordinates collected during the survey, I calculated the closest distance of each household to these shorelines using ArcMap (see Fig. S4 for an illustration). Taking the difference between the distance in January 2021 and the distance in January 2020 yielded a quantitative estimate of the objective household-level erosion extent in 2020 (and accordingly for the erosion extent of the years 2016 to 2019). I use January images since January is the first month after the monsoon for which cloud free optical images are available reliably for all years.

Data on temperature and precipitation was extracted from the Climatic Research Unit gridded Time Series (CRU TS), a widely used climate dataset on a 0.5° latitude by 0.5° longitude grid over all land domains of the world (Harris et al. 2020). Monthly data was collected for the period 1950–2020 for the five CRU grid cells which cover the 36 study locations.

### Perception data

Respondents assessed the occurrence of erosion at the riverbank closest to their house for the year preceding the survey (2020). To this end, I asked how far the river was from their house before the 2020 erosion and how far away it was at the time of the survey in June 2021.^8^ Calculating the difference between these distances allowed me to obtain a quantitative estimate of the respondents’ perceived erosion extent in 2020 (see Fig. S4 for an illustration). Erosion estimates below 0 (3.2% of the erosion estimates) or above 5000 m (1.2% of the estimates) were replaced with missing prior to the analysis due to a lack of plausibility. In addition to the absolute extent of erosion, respondents also stated whether, in their opinion, erosion had increased, not changed, or decreased over the five years preceding the survey.^9^

I estimated individual-level perception accuracy by calculating the difference between the satellite-based and the perceived erosion extent. Given that most other studies assess respondents’ perceptions of environmental change only through qualitative scales (Abid et al. 2019; de Longueville et al. 2020; M. K. Hasan & Kumar 2019; Moyo et al. 2012), my quantitative accuracy indicator represents a methodological advancement, which enables a more fine-grained analysis of which population groups perceive changes accurately and which do not.

I assessed respondents’ perceptions of temperature and dry- as well as wet-season precipitation in the past 20 years first in a binary way.^10^ Respondents who indicated that changes had occurred were then asked openly about the specific nature of these changes. The chosen time frame of 20 years is consistent with other studies of climate change perceptions (Bryan et al. 2009; de Longueville et al. 2020; Tambo and Abdoulaye 2013).^11^

In line with the theoretical expectations provided in Section 2, I include several individual-level covariates in the analyses: First, to assess the impact of the 2020 erosion, I included a question inquiring whether respondents had been personally affected by the respective erosion event. Second, I accounted for respondents’ occupations by recoding their main income source as either environmentally independent (0) or dependent (1). Third, I consider whether respondents have lived in the village since birth (1) or moved there at some point (0). Finally, I assess climate change literacy by whether respondents had heard of the term “climate change” (1) or not (0). Additional covariates include respondents’ age, education (on a six-point scale from 0 = “no education” to 6 = “university degree”) and sex, where I expect older and more educated respondents to perceive environmental changes more accurately due to their increased experience. In terms of sex, women have been found to perceive climatic changes more accurately than men (Shrestha et al. 2019).

To summarize, the main variables of interest are the respondents’ perceptions of long-term trends in temperature and precipitation (for the past 20 years) and erosion (for the past five years). For erosion, I additionally analyze the error of respondents’ perceptions for the year 2020, meaning the absolute difference between their perceptions and the measured erosion extent.

### Estimation strategy

Different regression models are estimated to assess which factors influence the respondents’ environmental perceptions. First, the magnitude of the respondents’ error in perceiving the 2020 erosion is modelled by a linear regression, including as explanatory variables the measured erosion extent and respondents’ distance from the riverbank, as well as the covariates outlined in Section 3.4 (erosion affectedness, occupation type, duration of residency in the village, sex, age, education). Second, I estimate quantile regressions with the same explanatory variables to assess heterogeneous effects within the respondent sample. Lastly, I model the influence of socio-demographic variables on respondents’ temperature and precipitation perceptions through linear regressions. The dependent variables are five-point-scales from 1 (strong temperature/precipitation decrease) over 3 (no change/more erratic) to 5 (strong increase). Summary statistics of these three variables are shown in Table S2. For these models, occupation type, duration of residency in the village, climate change literacy, and sex, age, and education are included as explanatory variables. To account for unobserved heterogeneity between villages, additional models include village fixed effects as a robustness check. To account for potential correlation between respondents of the same village, standard errors are clustered at the village level. All analyses were conducted in R.

## Results

### Perception of past erosion

**Fig. 2:**
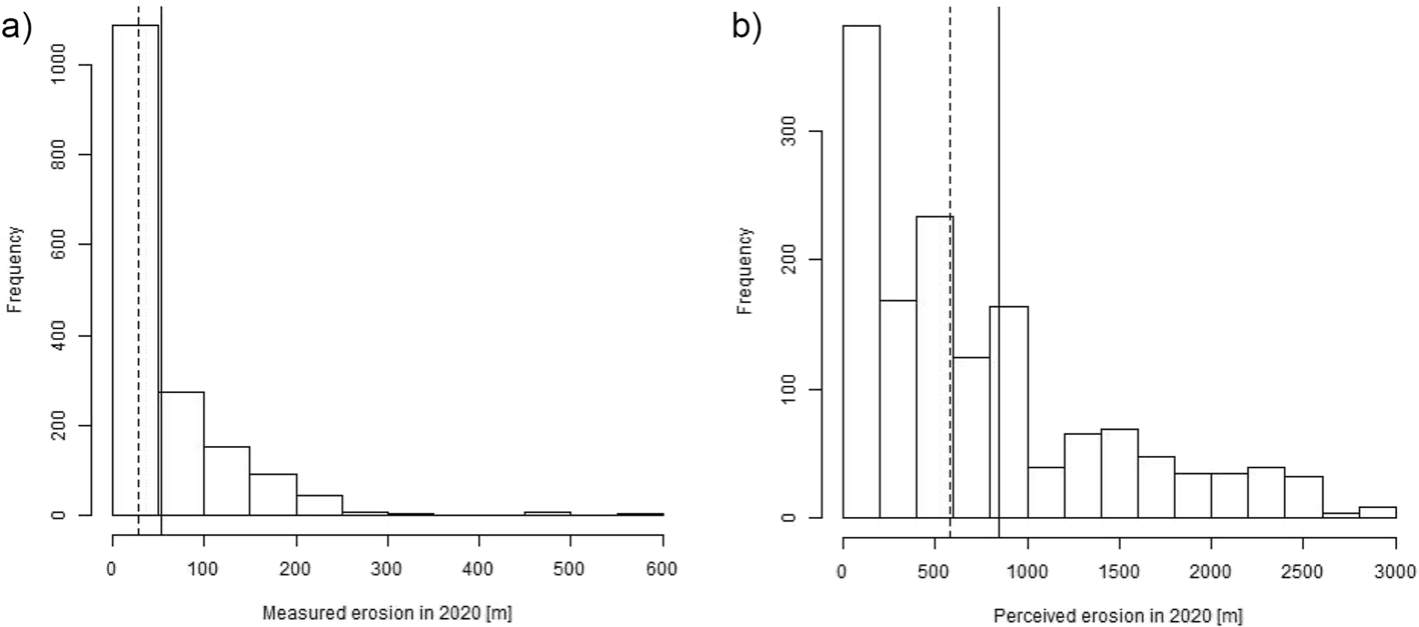
Distribution of (a) measured and (b) perceived (only values < 3000 m are plotted to enhance readability) erosion values for 2020. Vertical lines: median (dashed) and mean (solid)

Figure 2 presents the distribution of measured and perceived erosion values for the year 2020. Summary statistics of the main erosion-related variables are shown in Table S2. While the mean *measured* erosion is 53 m, the mean *perceived* erosion is 846 m and, hence, more than ten times higher. Regressing the measured on the perceived erosion extent yields a positive and highly statistically significant correlation (Table S3).

What is striking, however, is the size of the regression coefficient between the measured and perceived erosion variable (around 0.01). This implies that respondents perceive the river to erode around 100 times more land than it actually does. One possible explanation for this stark overestimation might be that respondents have difficulties estimating distances precisely. However, when I regress the measured distance between their house and the riverbank on the distance indicated by the respondents, I find a regression coefficient of 0.15 (see Fig. S2 and Table S4). Indeed, respondents overestimate the distance between their house and the riverbank by a factor of roughly 6.7. However, this means that their overestimation of the erosion extent (factor 100) can only partly be explained by difficulties in estimating distances.^12^ This suggests that the remaining overestimation is related to how respondents perceive the specific event of riverbank erosion.

**Fig. 3:**
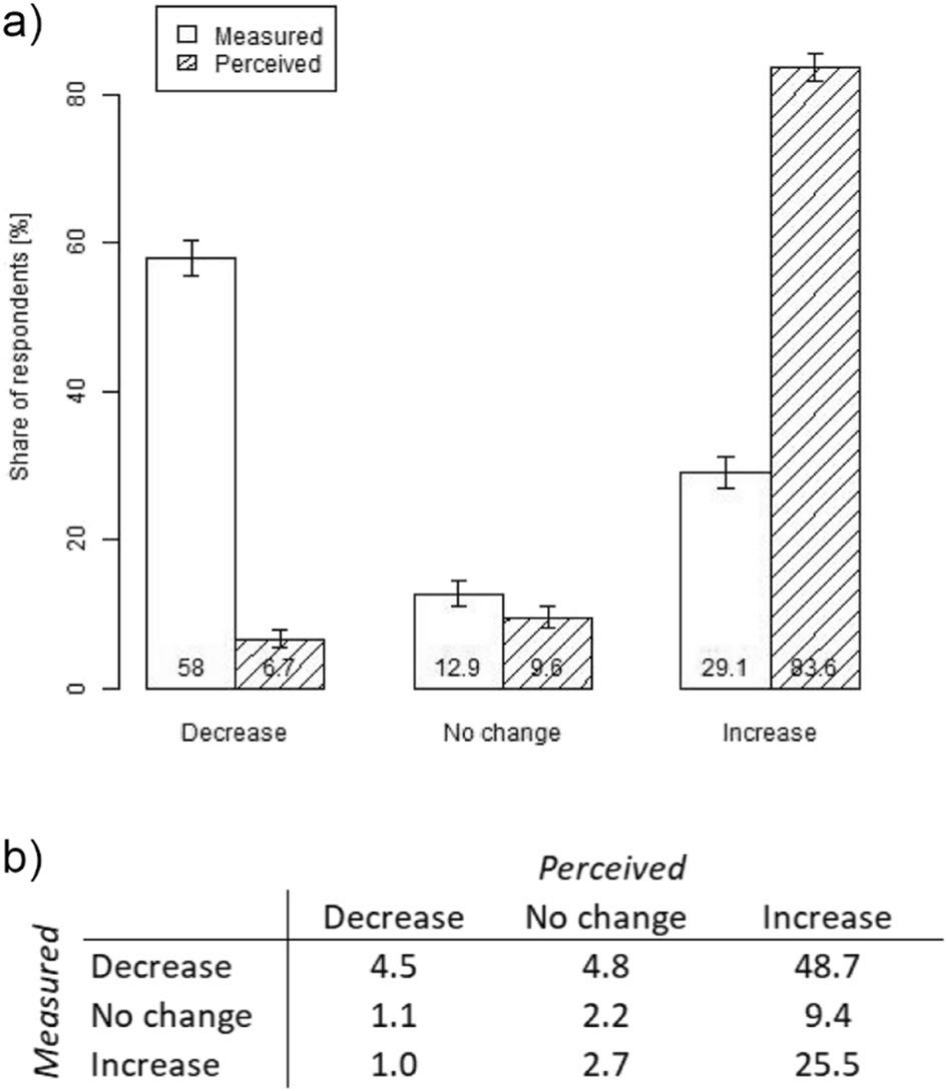
**a** Respondents’ perception of erosion changes in their home village in the past five years versus satellite-based measurements, with 95% confidence intervals. **b** Cross-tabulation of perceived versus measured changes in erosion (in percent)

Besides the absolute extent of erosion in 2020, respondents were asked whether they had perceived any changes in erosion over the past five years. Over 93% of the respondents felt that erosion had increased, while 10% and 7% perceived no change and a decrease, respectively (Fig. 3a). The satellite-based measurements, however, reveal that erosion had increased over the past five years only for 29% of the respondents, whereas it had not changed for 13% and it had decreased for 58%. Contrasting the individual-level perceptions and measurements, only 32% of the respondents perceive the erosion trend in the same direction as the measurements show (see diagonal in Fig. 3b). A significant share (49%) perceives an increase, while the measured erosion had in fact decreased. These findings about absolute levels and changes of erosion support hypothesis 1, which expects respondents to not perceive erosion accurately due to the underlying experiential processing.^13^

To investigate whether certain subgroups perceive erosion more accurately than others, I regress the error of the respondents’ erosion perception (defined as the absolute value of the difference between perceived and measured erosion) on several socio-economic variables (Table 1, see Table S2 and Table S5 for summary statistics and a correlation matrix of covariates, respectively). Respondents who were personally affected by erosion as well as those whose income depends on the environment, exhibit a significantly higher perception error than those who were neither affected nor dependent on the environment. These effects are also substantively relevant: Taking the intercept of 511 m as the baseline error, being affected by erosion increases the error by 42%, whereas an environmentally dependent income source increases it by 33%. Further, the magnitude of the error is correlated directly to the distance of a respondent’s house from the river: An additional distance of 100 m increases the perception error by 98 m. Respondents who were born in the village show a lower error than those who had moved to the village at some point in the past. Lastly, the objectively measured erosion extent does not have a significant influence on the size of the error. Except for “born in village,” these effects are robust to the inclusion of socio-demographic variables (model 1 in Table S6). With respect to the socio-demographic variables, male respondents show a significantly and substantively lower error than female ones. Age and education do not exert a significant influence on the perception error. Appendix C contains robustness checks and additional analyses to characterize particularly inaccurate respondents.

**Table 1:** Linear regression model of determinants of the magnitude of respondents’ error in perceiving the 2020 erosion

	*Dependent variable:*
	Error 2020 (m)
Eros. extent 2020 (m)	0.74
	(0.49)
Eros. impact 2020 (n/y)	216.44^***^
	(63.19)
Dist. from river (m)	0.98^*^
	(0.54)
Income env.-dep.? (n/y)	167.63^***^
	(52.84)
Born in village? (n/y)	-101.17^*^
	(53.88)
Intercept	510.63^***^
	(81.65)
Village FE?	No
Observations	1,348
R^2^	0.04
Adjusted *R*^2^	0.04
Residual Std. error	872.00 (df = 1342)
F Statistic	11.83^***^ (df = 5; 1342)

^*^p < 0.1; ^**^p < 0.05; ^***^p < 0.01

Standard errors clustered by village. (m) – (meters), (n/y) – (no/yes)

The abovementioned effects relate to the influence of the variables averaged across the entire sample. Effects might, however, differ for different parts of the sample, e.g., for those who show a very large error compared to those whose error is close to zero. In the present case, this might be especially relevant due to the long tail of the error variables (see Table S2 and Fig. S5).

Indeed, the results of the quantile regressions presented in Table 2 suggest non-linear effects. The parts of the population with the highest perception error (models 7, 8, and 9) are particularly strongly influenced by whether they have themselves been affected by erosion, by an increasing distance from the river, by having an environmentally dependent income source, and by not being born in the village. Except for “born in the village,” these effects also hold for those respondents with the lowest error (models 1, 2, and 3). In addition, the objectively measured erosion extent has a consistently significant and positive effect on those respondents. These effects are robust to the inclusion of socio-demographics (Table S7).

**Table 2:** Quantile regressions of the absolute error of the perception of the 2020 erosion extent. Model numbers correspond to the deciles (e.g., model 1 corresponds to the 10th percentile, model 2 to the 20th percentile, etc.)

*Dependent variable*									
Error (2020)									
	(1)	(2)	(3)	(4)	(5)	(6)	(7)	(8)	(9)
Eros. extent 2020 (m)	0.77^***^	0.85^***^	0.91^***^	0.93^***^	1.43^***^	1.43^***^	1.17^***^	0.36	-0.64^***^
	(0.09)	(0.23)	(0.21)	(0.30)	(0.37)	(0.36)	(0.30)	(0.56)	(0.20)
Eros. impact 2020 (n/y)	15.00	102.03^***^	135.83^***^	176.95^***^	231.71^***^	251.72^***^	300.59^***^	398.33^***^	339.69^**^
	(11.40)	(22.57)	(24.02)	(31.74)	(43.00)	(50.95)	(74.59)	(102.32)	(136.22)
Dist. from river (m)	0.13	0.60^***^	0.80^***^	0.97^***^	1.26^***^	1.21^***^	1.38^***^	1.89^**^	1.99^***^
	(0.09)	(0.19)	(0.18)	(0.26)	(0.30)	(0.31)	(0.51)	(0.74)	(0.55)
Income env.-dep.? (n/y)	8.05	47.91^**^	91.41^***^	111.54^***^	137.98^***^	201.85^***^	261.06^***^	238.50^**^	353.58^***^
	(6.12)	(23.36)	(24.36)	(32.28)	(43.59)	(51.15)	(75.44)	(104.27)	(118.85)
Born in village? (n/y)	-0.71	-30.76	-57.73^**^	-90.20^***^	-100.73^**^	-182.12^***^	-204.30^***^	-278.90^***^	-348.08^***^
	(6.50)	(23.40)	(25.53)	(33.49)	(44.44)	(54.93)	(78.90)	(102.65)	(109.94)
Intercept	-11.86^**^	-5.69	44.47	124.69^***^	172.25^***^	360.58^***^	539.58^***^	913.98^***^	1,590.51^***^
	(4.99)	(26.93)	(30.59)	(40.64)	(55.27)	(68.32)	(96.79)	(132.29)	(171.68)
Observations	1,352	1,352	1,352	1,352	1,352	1,352	1,352	1,352	1,352

^*^p < 0.1; ^**^p < 0.05; ^***^p < 0.01

(m) – (meters), (n/y) – (no/yes)

### Perception of temperature change

**Fig. 4:**
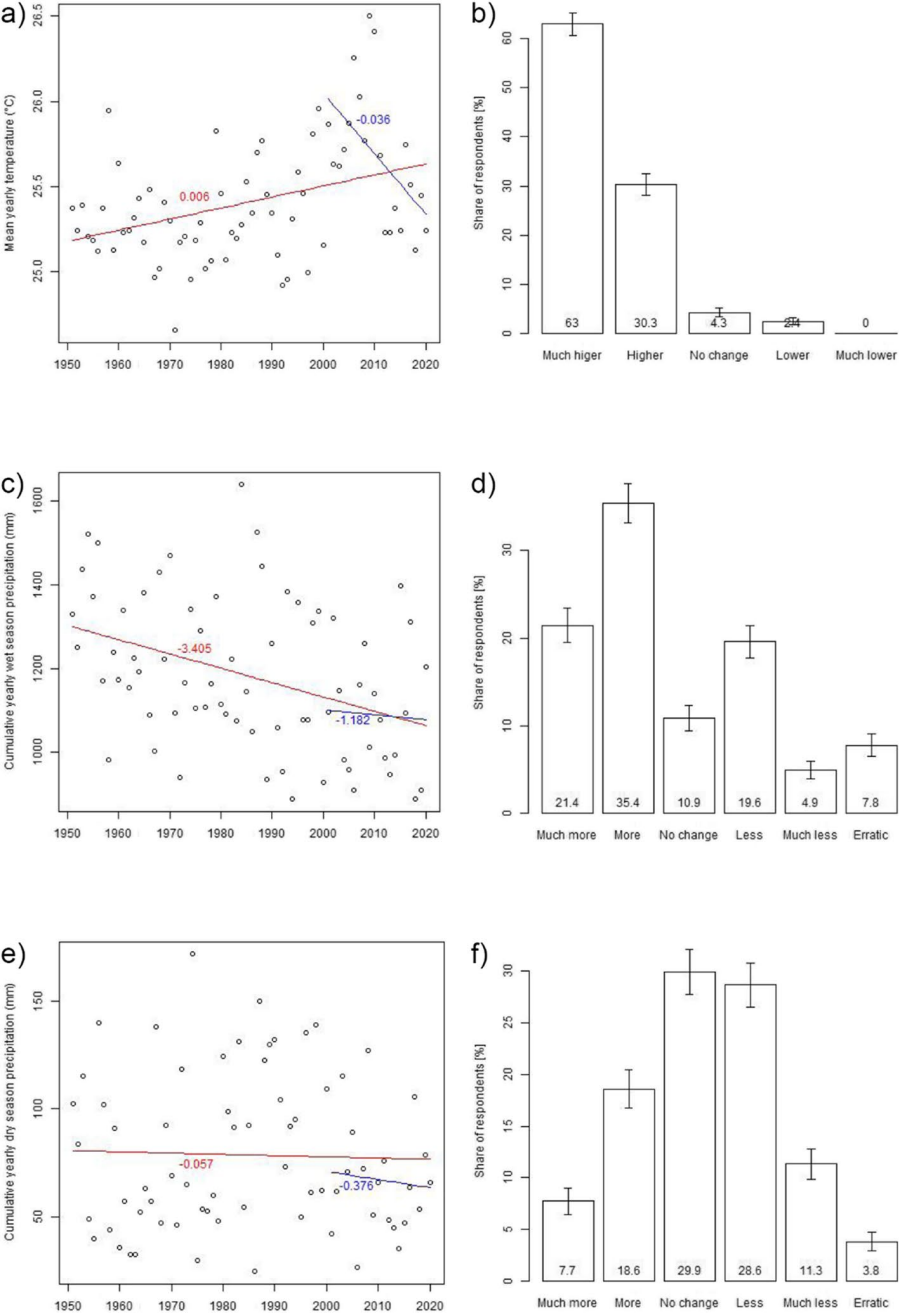
Left: mean yearly temperature (**a**), wet season (**c**), and dry season (**e**) precipitation in the study region between 1950 and 2020, including trend lines for the whole period (red) and for the past 20 years (blue line). Right: respondents’ perception of changes in temperature (**b**), wet season (**d**), and dry season (**f**) precipitation changes in their home village in the past 20 years with 95% confidence intervals

Based on the CRU data, the mean yearly temperature in the study region increased by, on average, 0.006 °C/year for the period 1951 to 2020 (Fig. 4a). Calculating the trend line for the period about which respondents were asked, i.e., 2001–2020, the temperature decreased by on average 0.036 °C/year. This negative slope of the short-term trend line is caused by the extraordinarily hot decade 2001–2010 (Fig S5a). Comparing recent temperatures to a longer time horizon, 15 out of 20 years between 2001 and 2020 were warmer than the mean temperature between 1951 and 1990 (Fig. S7a). The increase in the mean yearly temperature is reflected by a relatively uniform increase of monthly temperatures (Fig. S8a). Except for April and May, the temperature of all months increased between 0.35 and 0.5 °C. This implies that there is not a particular season for which the temperature increased more strongly than for other seasons.

The temperature data discussed above refer to the mean of the entire study region, i.e., the average temperature across the five grid cells of the CRU dataset into which the 36 study villages fall. However, the results remain largely unchanged when examining the five regions separately (Fig. S9): While the absolute temperature level is different between regions (decreasing from south to north, see Table S8), the trend lines between 1951 and 2020 have a consistently positive slope of comparable magnitude, while they are consistently negative between 2001 and 2020 (Table S9). Since there are no major differences between regions, the following analysis considers only the average temperature across all five CRU regions.

In terms of respondents’ perceptions of temperature changes between 2001 and 2020, 2% perceived a decrease, 4% perceived no change, and 93% perceived an increase (Fig. 4b). Comparing these perceptions to the temperature trend between 2001 and 2020, only 4% of the respondents correctly perceived the negative temperature trend as measured for the past 20 years. Given, however, that the long-term trend between 1950 and 2020 is significantly increasing and hence opposite to the 20-year-trend, it is difficult to understand whether respondents restricted themselves to only the past 20 years when reflecting about temperature changes, or whether their perceptions were also influenced by the long-term trend. In the latter case, 93% would have correctly detected the overall increase in temperature over the past decades.

### Perception of wet-season precipitation

Between 1951 and 2020, the yearly wet-season precipitation (defined as the cumulative precipitation between June and August) declined by, on average, 3.4 mm/year (Fig. 4c; see also Fig. S6b, Fig. S7b and Fig. S8b). Considering only the last 20 years, the wet-season precipitation declined as well, albeit at a lower rate and not statistically significant. As for temperature, absolute values of wet-season precipitation differ between the five CRU regions that comprise the study area (increasing from south to north, see Table S10), but the described trends occur in a comparable way in each of the regions (Fig. S10 and Table S11).

While 11% of the respondents perceived no change in wet-season precipitation, 57% felt an increase, 25% a decrease, and 8% perceived the precipitation to be more erratic (Fig. 4d). Comparing these perceptions to the measured trend lines, 25% of the respondents correctly perceive the long-term decline in wet-season precipitation. Given, however, that the trend of the last 20 years is insignificant and that the variation between years is large, also the perceptions of those respondents who felt no change or that precipitation had become more erratic can be considered in line with the meteorological evidence. The remaining 57% of the respondents who perceived an increase are clearly not aligned with the objective data.

### Perception of dry-season precipitation

The yearly dry-season precipitation (defined as the cumulative precipitation between November and March) does not exhibit a significant trend for the period from 1951 to 2020 nor between 2001 and 2020 (Fig. 4e). However, the decadal trend (Fig. S6c), the deviation of the yearly dry season precipitation from the mean yearly dry season precipitation for the period from 1951 to 1990 (Fig. S7c) as well as the deviation of the mean monthly precipitation for the period from 2001 to 2020 from the mean monthly precipitation for the period from 1951 to 1990 (Fig. S8b) suggest a decline in dry-season precipitation. In particular, November and March experience, on average, lower rainfall than between 1951 and 1990, while the remaining dry-season months are largely unchanged.

Considering the five CRU regions, absolute values of dry-season precipitation decrease from south to north (Table S12). As for the average across the five regions, trend lines within the five regions are insignificant (Fig. S11 and Table S13). 30% of the respondents correctly perceive that dry-season precipitation has not changed across the past 20 years (Fig. 4f). Given the slightly decreasing (albeit insignificant) trend of the past 20 years and the large inter-annual variation, also the perceptions of those respondents who felt a decrease (40%) or more erratic precipitation patterns (4%) can be considered in line with the meteorological evidence. This implies that only the perceptions of those respondents who felt an increase in dry season precipitation (26%) stand clearly in contrast to the objective data.

### Determinants of climate change perceptions

Besides assessing the overall accuracy of temperature and precipitation perceptions, I model the influence of socio-demographic variables on respondents’ perceptions (Table 3). I find that having an environmentally dependent occupation is associated with a significantly lower perception of temperature increase and a significantly higher perception of wet-season precipitation increase compared to occupations which are independent of the environment.

**Table 3:** Determinants of respondents’ perception of temperature, wet and dry season precipitation changes (linear regressions). The dependent variables are five-point-scales from 1 (strong temperature/precipitation decrease) over 3 (no change/more erratic) to 5 (strong increase)

	*Dependent variable:*		
	Temperature	Wet season precip	Dry season precip
	(1)	(2)	(3)
Income env.-dep.? (n/y)	− 0.19^***^	0.10^*^	− 0.01
	(0.04)	(0.06)	(0.06)
Born in village? (n/y)	− 0.03	− 0.005	0.05
	(0.04)	(0.06)	(0.06)
Heard of climate change? (n/y)	0.11^**^	− 0.11^*^	− 0.04
	(0.05)	(0.06)	(0.06)
Intercept	4.63^***^	3.44^***^	2.80^***^
	(0.07)	(0.06)	(0.06)
Village FE?	No	No	No
Observations	1,456	1,436	1,418
R^2^	0.03	0.005	0.001
Adjusted R^2^	0.03	0.003	− 0.001
Residual Std. Error	0.66 (df = 1452)	1.15 (df = 1432)	1.09 (df = 1414)
F Statistic	14.98^***^ (df = 3; 1452)	2.26^*^ (df = 3; 1432)	0.34 (df = 3; 1414)

^*^p < 0.1; ^**^p < 0.05; ^***^p < 0.01

Standard errors clustered by village. (n/y) – (no/yes)

Respondents who had already heard the term “climate change” have a significantly higher perception of temperature increase and a significantly lower perception of wet-season precipitation change than those who had not heard it.^14^ However, this does not mean that only those respondents who had already heard about climate change perceived temperature increases. Among the 965 respondents (62% of the sample) who had not heard the term “climate change”, 93% perceived a temperature increase, while only 4% perceived no change and 3% perceived a decrease.

As robustness checks, Table S14 presents models including socio-demographics (sex, age, education) as well as village fixed effects. The effects of income source and climate change literacy on temperature change perceptions appear robust across model specifications. The effects on wet season precipitation perceptions are robust to the inclusion of socio-demographics but become insignificant when adding village-fixed effects. They hence appear less robust than those on temperature perceptions.

Overall, it appears remarkable that there are no consistent patterns between the three climatic indicators. Even those variables that have a significant influence have a positive influence on one indicator but a negative one on another indicator. As for the accuracy of perceptions discussed in previous sections, it seems that the influence of socio-demographics on climate change perceptions depends on the specific indicator considered.

## Discussion and conclusions

This paper investigates how accurately different environmental and climatic changes are perceived by a rural population in Bangladesh and which factors influence the accuracy of their perceptions. With respect to riverbank erosion, I find a high correlation between objectively measured and perceived erosion extent. However, the extent was overestimated by a factor of around 100, and it appears that this strong overestimation is driven by the specific nature of the erosion event. In particular, the Jamuna is a highly dynamic river that does not only erode a lot of land each year but also forms new land. In addition, it comprises multiple channels, some of which run dry during the dry season. Hence, the river system changes starkly from year to year, but also within the course of a single year. This leads to a lack of permanent landmarks, which might make it difficult for respondents to give a precise estimate of the absolute extent of erosion. However, also the erosion trend over the past five years was misperceived by most of the respondents. Since it is a qualitative estimate (in-/decrease), it should be easier to assess than absolute levels of erosion. The fact that the trend was also overestimated by most respondents supports my argumentation, expecting respondents to perceive erosion inaccurately due to the experiential processing of local extreme events.

Further, I find that it is mainly those respondents who have been personally affected by erosion that are particularly prone to misperceive erosion. This confirms the argument of Osbahr et al. (2011), who present an alternative explanation of why different climatic indicators are perceived with different levels of accuracy. Instead of the mechanism of psychological processing, they argue that it is the impact on respondents’ livelihoods that drives environmental perceptions: While two years t and t + 1 might have very similar meteorological characteristics; still, they might be perceived differently if a household’s vulnerability changes in between the two years.

In the present study, I find evidence supporting the importance of taking livelihood impacts into account. In my case study region, erosion has profound effects on respondents by destroying their farmland and houses. Such drastic impacts might evoke a strong emotional, affective reaction, which might ultimately bias their recall of the actual erosion extent. Besides those directly affected by erosion, respondents whose income depends on the environment are more likely to overestimate the extent of erosion.^15^ Given the high importance of favorable environmental conditions for their livelihoods, these respondents might develop a tendency to overestimate the changes that have occurred. This result contradicts previous studies, which either found that climate-sensitive households have a higher level of accuracy than non-sensitive ones (Kosmowski et al. 2016; Shrestha et al. 2019) or could not find differences in perceptions between respondents with or without agricultural jobs (Linke et al. 2020).

Regarding climatic factors, the measured long-term increase in mean annual temperature was perceived by 93% of respondents.^16^ For precipitation, by contrast, 57% (wet season) and 26% (dry season) misperceived the trend observed in the climate data. Considering the climate data for my study region, one possible explanation for why temperature is perceived more accurately might be that the inter-annual variation of precipitation is much larger than that of temperature (see Fig. S7). Further, average precipitation has increased for some months of the year, while it has decreased for others, whereas the average temperature has increased for all months (see Fig. S8). These factors might make it harder to detect long-term trends of precipitation change.

Similar to my findings, other studies have found that rural populations in Bangladesh perceive temperature changes accurately (Alam et al. 2017; Hasan and Kumar 2019). By contrast, these studies also concluded that precipitation changes are felt accurately, which the present study can only partly confirm. However, my findings that temperature changes are perceived more accurately than precipitation changes are in line with a large body of literature from various country contexts (see Section 1). Other studies from Bangladesh conclude that rural populations perceive changes in both temperature and precipitation but do not compare these perceptions to meteorological evidence (Alam and Mallick 2022; Kabir et al. 2016; Uddin et al. 2017). Regarding extreme events (erosion and floods), different studies discuss perceptions of riverine populations in Bangladesh without, however, comparing these perceptions to an individual-level, measured baseline (Alam et al. 2017; Das 2011; Hasan and Kumar 2019). Hence, this study is the first to analyze the individual-level accuracy of erosion perceptions in the Bangladeshi context. It is worth noting that riverbank erosion occurs not only along the Jamuna River and other major rivers in Bangladesh but also along various major rivers worldwide (e.g., Mekong River, Yellow River, Mississippi River, or Danube River), making the findings of this study relevant beyond the specific case study of Bangladesh.

With respect to climate change literacy, only 38% of the respondents of this study were familiar with climate change, whereas Haq and Ahmed (2017) found a much higher value of 83% among a rural Bangladeshi population. Interestingly, their study found a similar pattern of perceived reasons for climate change and erosion as the present study: Over 50% of the respondents of my study attribute climate change and erosion to the wish of God, whereas 25% think it is a natural phenomenon and the remaining respondents believe it is partly or fully human-induced. Whether and how such a strong spiritual perspective on the environment influences perceptions of environmental changes and risks as well as adaptation intentions might be a worthwhile undertaking for future research. Lastly, it is noteworthy that even remote populations that are hardly reached by educational measures perceive the climate to be changing. These findings contradict the argument that we only “feel” climate change because we cognitively know it exists (Howe 2018; Howe and Leiserowitz 2013).

Overall, my findings on erosion perceptions imply that caution is needed for survey research relying solely on respondents’ perceptions of sudden-onset environmental changes. Especially in settings where these changes have profound impacts on respondents’ livelihoods, it appears that there is a tendency to overestimate their extent. For research that requires precise estimates of environmental changes, survey-based perception data should ideally be complemented by objectively measured data. Still, this should not imply that subjective data is useless and should be replaced by objective measurements. Human actions are shaped by their perceptions, so even if these perceptions are inaccurate with respect to an objective baseline, they cannot be neglected if the goal is to understand why humans behave in certain ways. In any case, the present study is the first to assess the accuracy of perceptions of sudden-onset environmental changes. Equivalent analyses for other types of environmental events and for other geographical settings are required to consolidate our understanding of how people perceive such events.

In terms of wider policy relevance, the stark overestimation of the erosion extent and trend bears the danger of maladaptation. As Koubi et al. (2022) show, perceptions of riverbank erosion can significantly increase migration aspirations, meaning a belief that moving away is preferable to staying in situ. While migration can be a viable adaptation strategy (Afifi et al. 2016; Black et al. 2011; Vinke et al. 2022), it might be inappropriate if it is based on an overly extreme perception of environmental changes. Governments might hence aim at decreasing such misperceptions, e.g., by targeting communication strategies specifically at those parts of the population who are most at risk of overestimating the occurring changes (in the present case, this might be environmentally dependent households).

### Supplementary Information

Below is the link to the electronic supplementary material.

Supplementary file1 (PDF 2.23 MB)

## Data Availability

The datasets generated and analyzed during the current study are available in the Zenodo repository, 10.5281/zenodo.7633448

## Footnotes

1 Environmental perceptions comprise “both direct experience with environmental events (…) and indirect information from other people, government agencies, scientists, the mass media, and also fellow community members” (Koubi et al. 2016).

2 Perceptions cannot be “wrong” since they are social constructs (Stehr and Storch 1995). However, they can have a low correlation with objectively measured data, which is how I understand the term “accuracy” throughout this paper.

3 Note that climatic changes are a specific type of environmental changes. The latter also comprise non-climate-related changes such as geological processes (e.g., earthquakes). Studying both climatic and environmental changes is important to gain a holistic understanding of environmental perceptions.

4 Erosion processes are particularly suited for assessing perception accuracy for rapid-onset events since they can be more easily quantified objectively (e.g., using satellite imagery) than other discrete events such as floods or storms.

5 While in the survey, I ask respondents only about long-term changes and not about extreme events such as heat waves or extreme rainfall events, it cannot be excluded that such events influence respondents’ perceptions of long-term trends.

6 While it could be argued that the mere fact of living in a village that was severely affected by erosion evokes affect even if someone was not affected personally, it seems evident that personal affectedness will cause an even stronger affective reaction. Hence, I focus on personal affectedness.

7 High response rate, random GIS sample of households within villages, and surveying of almost all inhabited survey locations with erosion risk along the left bank of the 250 km of the Jamuna River.

8 While there is a temporal lag between the satellite image used for the objective erosion estimate (January) and the time of the survey (June), this should not affect results since erosion occurs primarily between June and October and is minimal during the dry season.

9 “In your opinion, has the riverbank erosion changed over the last 5 years in the location of your village?”.

10 Specifically, I asked: “In your opinion, has the temperature changed over the last 20 years in the location of your village?” and «In your opinion, has the amount of rain during the monsoon / during the dry season (November to March) changed over the last 20 years in the location of your village?”.

11 Even though a period of 20 years might induce recall bias, there is no methodological alternative for assessing respondents’ perception of long-term climatic changes.

12 Also, it cannot be explained by respondents being unfamiliar with the unit “meters”, since they could answer in whatever distance unit they were familiar with, and answers were only a posteriori converted to meters.

13 Note that these inaccurate perceptions cannot be explained by survey effects (meaning that the large number of survey questions asked about erosion made respondents overestimate the change of erosion in the past five years), since the question about changes in erosion was the first time that erosion was mentioned in the questionnaire.

14 Note that I assessed respondents’ familiarity with the concept “climate change” only after asking about their perceptions of temperature and precipitation.

15 These effects were, however, not fully robust across model specifications (e.g., they hold when controlling for education, but become partly insignificant when including village fixed effects). While the underlying mechanism is not fully clear, it might hint towards a third variable that is related to both respondents’ occupation type and their perception accuracy, and that is not included in the models.

16 Note that the assessment of erosion perceptions is much more fine-grained given that I asked about the precise extent of erosion, whereas temperature and precipitation changes were assessed only qualitatively (in-/decrease).
