# Supplementary material for: Perceptions of environmental changes among a climate-vulnerable population from Bangladesh
Source: Clim Change. 2024 Feb 1;177(2):25. doi: 10.1007/s10584-024-03678-6 (PMC10830697; doi:10.1007/s10584-024-03678-6)
Supplement: Supplementary file 1 — Supplementary file1 (PDF 2.23 MB) [file 10584_2024_3678_MOESM1_ESM.pdf]

Supplementary Information

Perceptions of environmental changes among a climate-vulnerable population from Bangladesh

Jan Freihardt[1]

[1]Center for Comparative and International Studies (CIS), ETH Zurich, 8092 Zurich, Switzerland

Correspondence to: Jan Freihardt (jan.freihardt@ir.gess.ethz.ch), ORCID: 0000-0003-2096-0335

Journal: Climatic Change, DOI: 10.1007/s10584-024-03678-6

Appendix A: Selection of study locations

I selected participants in a multi-stage cluster design. In the first stage, I selected 36 locations along the easternmost riverbank line of the Jamuna because the rates of riverbank erosion are higher along the eastern than along the western riverbank due to differences in floodplain materials (CEGIS, 2018; Sarker et al., 2014). I identified this line using the most recent satellite imagery available. Villages on chars (sandy islands in the river) were not considered since char populations have adapted their livelihoods to the yearly recurring flood and erosion events (Alam et al., 2017; Islam et al., 2015).

Along this line, I defined 250 sampling points with a one-kilometer distance along the whole stretch of the river (from the border with India in the north to the convergence of Ganges and Jamuna in the south). Ideally, I would have drawn the 36 study locations randomly from this pool of 250 stretches. However, a visual, satellite-based analysis revealed that not all of these 250 stretches were suitable for my study purpose. Therefore, I evaluated each of the 250 stretches with respect to the following two criteria: First, survey feasibility, that is, whether there were enough settlements (= at least 75 houses) in the 200 m stretch inland; and second, the ex-ante risk for riverbank erosion, in particular, whether there was a clear indication of a permanent embankment structure that prevents erosion and whether there was a char/large sandbank in front of the stretch that blocks erosion.

Stretches for which the satellite analysis showed that at least one of these criteria was violated were excluded from the pool. This reduced the pool size from 250 to 79 stretches. For some of these 79 stretches, not all criteria could be clearly evaluated from the satellite images due to insufficient image resolution. Therefore, the final screening was done on the ground during a field visit. Six stretches could not be visited due to their remote location. Of the remaining 73 stretches, 29 were excluded after the field visit due to a violation of at least one of the three criteria. One stretch was used for training the enumerators, leaving 43 stretches suitable for my sample. Due to time constraints during the fieldwork, not all 43 stretches could be included in the sample. Therefore, I chose 36 stretches such that they were well distributed along the entire length of the Jamuna. An overview of the 36 locations is provided in Fig. S1. Table S1 provides a list of all 79 stretches initially in the sample, including whether they were part of the final sample and – if not – the reason for their exclusion.

At each of the 36 locations, households were sampled using a stratified random spatial sampling design to survey households located within three zones defined by distance from the shoreline. This design allowed capturing potential effects of different ex-ante erosion risk levels on perceptions of environmental changes. At each location, the three zones were constructed by shifting the shoreline inland by 50 m, 100 m and 200 m, respectively. Consequently, each sampling zone has an extent of 1 km in the direction of flow and of 200 m inland.

Within each of the three zones, a spatially explicit sample was generated following the procedure outlined by Crawford et al. (2020). Specifically, a set of 25 random latitude-longitude points per zone was created using ArcMap software (with a minimum distance of 10 m between points). In the field, enumerators navigated to these points using smartphones. Having arrived at the point, they selected the house closest to that point based on visual estimation. This household was subsequently interviewed (see Fig. S2 for the distribution of the households' distance from the riverbank). If a household declined participation or if the household head was not available at two contact attempts,

the enumerator continued to the next closest household, in reference to the starting point. Within each household, the household head was interviewed, defined as the decision-maker within the household.

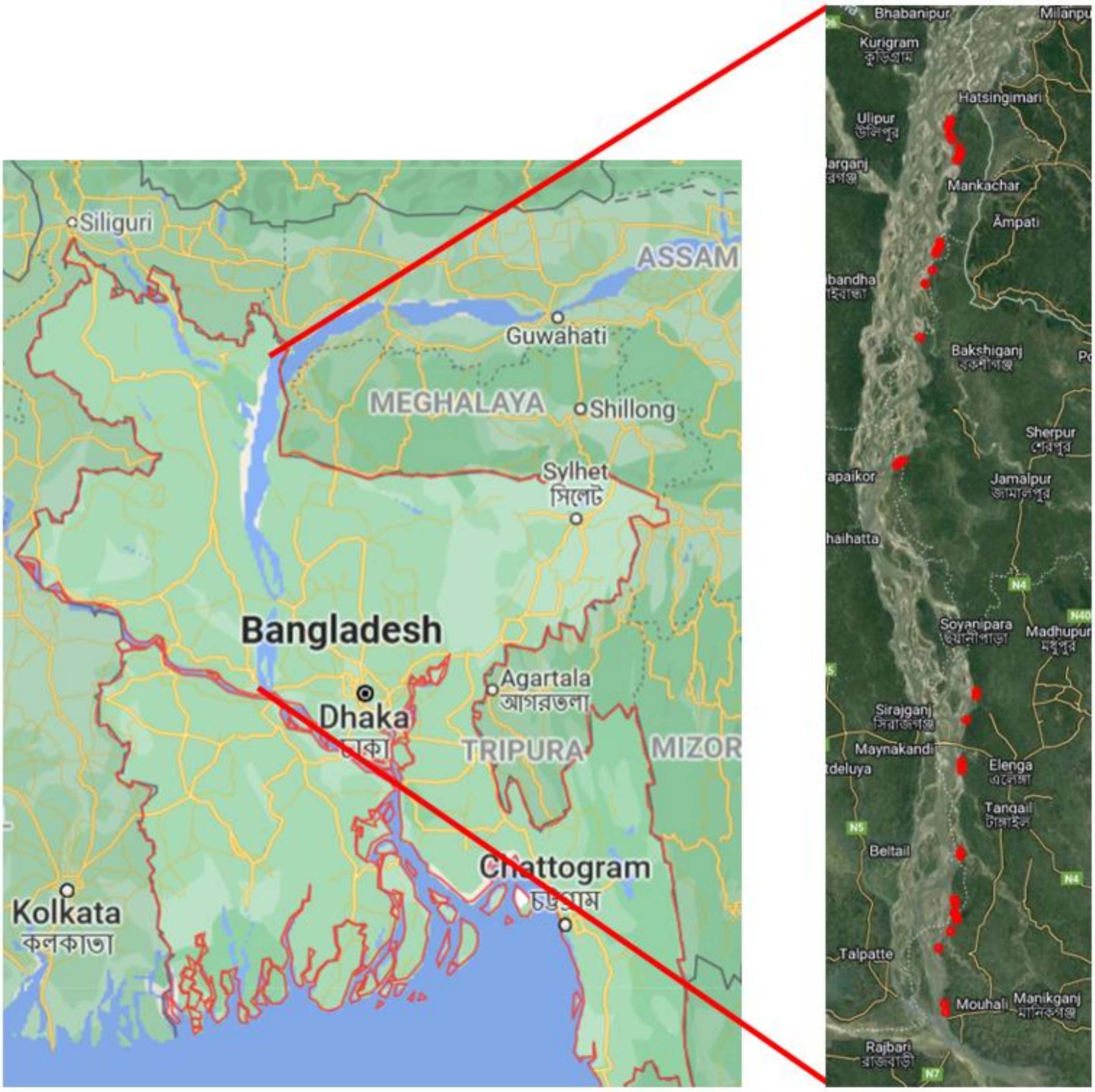

Fig. S1 Overview of the 36 study locations. Copyright map: Google. Copyright satellite image: TerraMetrics, 2022

Table S1 List of 79 stretches initially in the sample

Site	Latitude	Longitude	District	Sampled	Reason for exclusion
1	23.84858386	89.77728293	Manikganj	Yes	
2	23.85741259	89.7761692	Manikganj		Suitable, excluded for time constraints
3	23.8659592	89.77336891	Manikganj	Yes	
4	23.8741437	89.7696434	Manikganj		Training site
5	23.88306306	89.76938992	Manikganj		Suitable, excluded for time constraints
6	23.89188075	89.77113454	Manikganj		Embankment
7	23.93977265	89.77300493	Manikganj		Not visited
8	23.96265272	89.75980911	Manikganj		Char/sandbank
9	23.971464	89.75801152	Manikganj		Char/sandbank
10	23.97946232	89.76071457	Manikganj	Yes	
11	23.98646574	89.76636763	Manikganj		Not enough settlement
12	23.99336663	89.77212095	Manikganj		Not enough settlement
13	24.00092974	89.77700303	Manikganj		Not enough settlement
14	24.0080967	89.78240531	Manikganj		Not enough settlement
15	24.01468743	89.78848452	Tangail	Yes	
16	24.01998107	89.79575917	Tangail		Not visited
17	24.02782786	89.79999907	Tangail		Not visited
18	24.03669272	89.80143637	Tangail	Yes	
19	24.04554666	89.80018357	Tangail	Yes	
20	24.05359151	89.79620979	Sirajganj	Yes	
21	24.06191619	89.79537304	Sirajganj		Not enough settlement; char/sandbank
22	24.07061319	89.79705522	Sirajganj	Yes	
23	24.07936755	89.79571167	Sirajganj	Yes	
24	24.08826802	89.79443957	Sirajganj		Not enough settlement; char/sandbank
25	24.09651555	89.79100124	Sirajganj		Char/sandbank
26	24.10416064	89.78625988	Sirajganj		Char/sandbank
27	24.11252358	89.78760715	Sirajganj		Not enough settlement; embankment
28	24.1199595	89.79245961	Sirajganj		Embankment
29	24.12686204	89.79824051	Sirajganj		Embankment
30	24.13388967	89.80382386	Sirajganj		Embankment
31	24.14197229	89.80777083	Sirajganj		Embankment
32	24.15055663	89.81028585	Tangail		Not enough settlement; embankment
33	24.15953767	89.81062441	Tangail		Suitable, excluded for time constraints
34	24.16836954	89.80947906	Tangail	Yes	
35	24.17727796	89.80949719	Tangail	Yes	
36	24.2295696	89.78659759	Tangail		Not visited
37	24.23854823	89.7860587	Tangail		Not visited
38	24.34224707	89.81162957	Tangail	Yes	
39	24.35117757	89.81254109	Tangail	Yes	
40	24.36017152	89.81221315	Tangail	Yes	
41	24.36889043	89.81040512	Tangail		Not enough settlement; embankment
42	24.38032687	89.8048785	Tangail		Embankment
43	24.43313402	89.8199736	Tangail		Char/sandbank
44	24.44195819	89.82141819	Tangail	Yes	

45	24.45047689	89.82397444	Tangail	Yes	
46	24.49471362	89.84519411	Tangail	Yes	
47	24.50358737	89.84590586	Tangail	Yes	
48	24.51181581	89.84245001	Tangail		Embankment
49	24.58706697	89.81975046	Jamalpur		Not enough settlement; embankment
50	24.59501471	89.81563181	Jamalpur		Not enough settlement; embankment
51	24.60315021	89.81184187	Jamalpur		Embankment
52	24.61077011	89.80728877	Jamalpur		Embankment
53	24.61961004	89.80620216	Jamalpur		Embankment
54	24.9010855	89.65554932	Bogra		Not visited
55	24.96551006	89.66537782	Bogra	Yes	
56	24.97114163	89.67177488	Bogra	Yes	
57	24.9745598	89.68009865	Jamalpur		Not enough settlement
58	24.98684177	89.69272319	Jamalpur		Not enough settlement
59	25.00071823	89.70405209	Jamalpur		Char/sandbank
60	25.21211443	89.72276084	Jamalpur		Not enough settlement
61	25.22079034	89.7204164	Jamalpur	Yes	
62	25.22963506	89.7196273	Jamalpur	Yes	
63	25.33554382	89.72866088	Gaibandha	Yes	
64	25.36243599	89.7450544	Jamalpur	Yes	
65	25.38816632	89.74946849	Kurigram		Suitable, excluded for time constraints
66	25.39617635	89.75357157	Kurigram	Yes	
67	25.40397791	89.75805218	Kurigram	Yes	
68	25.41161802	89.76276468	Kurigram	Yes	
69	25.42006097	89.76266573	Kurigram	Yes	
70	25.58524338	89.80014675	Kurigram	Yes	
71	25.59256635	89.80534785	Kurigram	Yes	
72	25.60067086	89.80889396	Kurigram	Yes	
73	25.60950232	89.80727078	Kurigram	Yes	
74	25.62799682	89.78911681	Kurigram	Yes	
75	25.63403951	89.78356735	Kurigram	Yes	
76	25.64292418	89.78384213	Kurigram	Yes	
77	25.65177817	89.78241922	Kurigram	Yes	
78	25.66053126	89.78367557	Kurigram	Yes	
79	25.66861671	89.78750184	Kurigram	Yes	

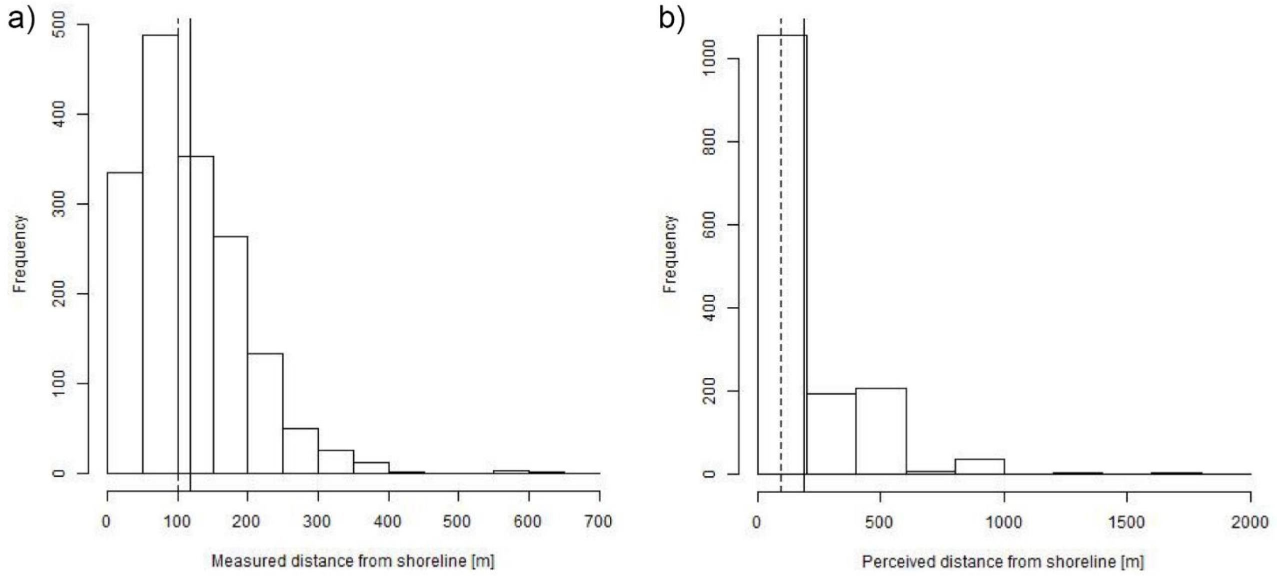

Fig. S2 a) Measured and b) perceived distance of households from next closest riverbank of Jamuna River. Vertical lines: median (dashed) and mean (solid)

Appendix B: Supporting figures and tables

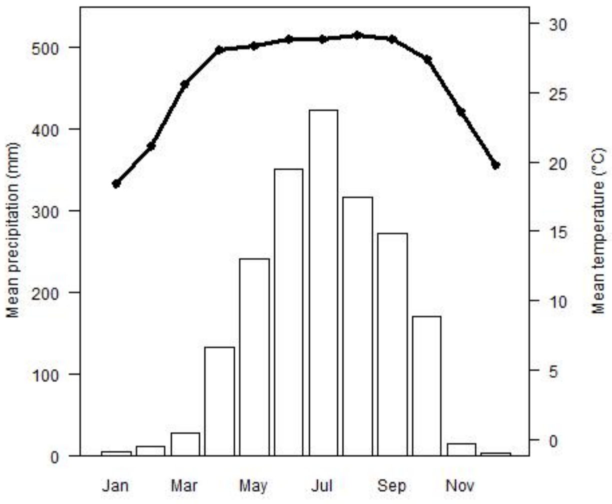

Fig. S3 Climate diagram of the study region. Bars: mean monthly precipitation, line: mean monthly temperature

Table S2 Summary statistics of co-variates, of variables of measured and perceived erosion and of respondents' perceptions of temperature and precipitation changes

Variable	N	Mean	Std. Dev.	Min	Pctl. 25	Pctl. 75	Max
Age	1695	47.58	14.01	18	37	57.5	115
Sex	1698	0.87	0.34	0	1	1	1
Education	1697	0.86	1.34	0	0	1	5
Income env.-dep.?	1617	0.56	0.5	0	0	1	1
Born in village?	1694	0.56	0.5	0	0	1	1
Heard of climate change?	1549	0.38	0.48	0	0	1	1
Distance to river 2021	1673	118.75	80.52	1.01	58.42	163.49	689.42
Erosion extent 2020	1673	53.16	71.16	0	0	79.63	591.46
Perceived erosion 2020	1495	845.88	900.48	0	191.44	1249.5	4998
Personal affectedness by erosion 2020	1629	0.59	0.49	0	0	1	1
Actual error 2020	1474	791.93	888.29	-341.56	166.6	1148.92	4945.98
Absolute error 2020	1474	799.26	881.69	0	166.6	1148.92	4945.98
Overestimator 2020	571	0.71	0.46	0	0	1	1
Perc. of temperature change	1683	4.54	0.69	2	4	5	5
Perc. of wet-season precip. change	1657	3.49	1.17	1	3	4	5
Perc. of dry-season precip. change	1639	2.83	1.09	1	2	4	5

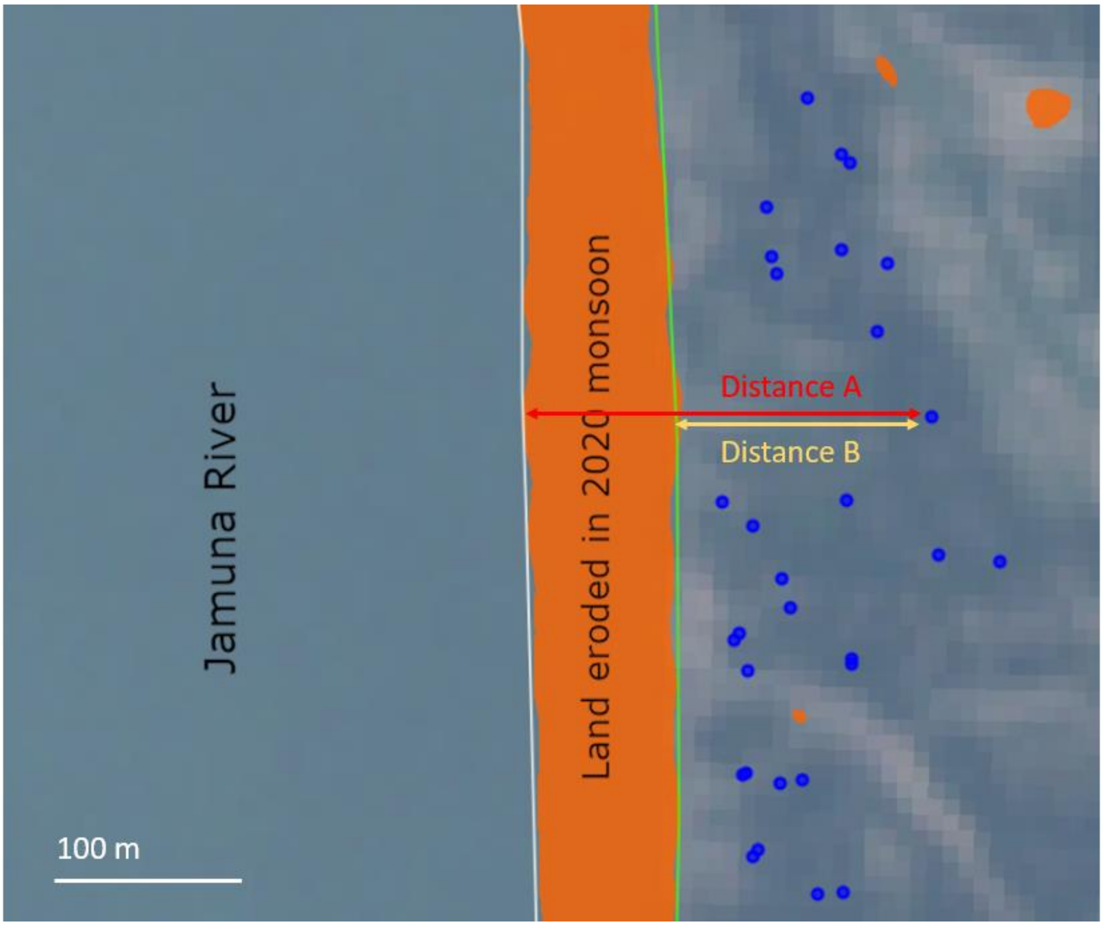

Fig. S4 Illustration of measured and perceived erosion estimation. Blue dots: locations of respondents' houses, coordinates were recorded during the survey. White and green line: bankline in January 2020 and January 2021, respectively. Orange area: land classified as "eroded in 2020" by the algorithm from Freihardt and Frey (2023). Distance A: **objectively** determined in ArcMap as the distance of the blue dot from the white line / **subjectively** estimated by respondents from the question "How far away from your house was the river before the 2020 erosion?". Distance B: **objectively** determined in ArcMap as the distance of the blue dot from the green line / **subjectively** estimated by respondents from the question "How far away from your house is the river now?". The difference between Distance A and Distance B yielded the measured / perceived extent of erosion during the 2020 monsoon season. Background image: Sentinel-2

Table S3 Regression of measured on perceived erosion extent

	Dependent variable:
	Erosion extent 2020
Perceived erosion 2020	0.01***
	(0.004)
Intercept	42.54***
	(9.10)
Observations	1,474
R^2	0.03
Adjusted R^2	0.03
Residual Std. Error	72.32 (df = 1472)
F Statistic	42.90*** (df = 1; 1472)
Note:	*p<0.1; **p<0.05; ***p<0.01
	Standard errors clustered by village.

Table S4 Regression of measured distance of respondents' houses to the riverbank on the distance indicated by the respondents

	Dependent variable:
	Measured distance 2021
Perceived distance 2021	0.15***
	(0.04)
Intercept	91.66***
	(6.19)
Observations	1,490
R^2	0.18
Adjusted R^2	0.18
Residual Std. Error	72.99 (df = 1488)
F Statistic	320.22*** (df = 1; 1488)
Note:	*p<0.1; **p<0.05; ***p<0.01
	Standard errors clustered by village.

Table S5 Correlation matrix of socio-demographic variables

Variable 1	Variable 2	Correlation coefficient	p-value
Age	Sex	0.10	5.59E-05
Age	Education	-0.20	0.00E+00
Sex	Education	0.04	7.91E-02
Age	Income env.-dep.?	0.06	1.27E-02
Sex	Income env.-dep.?	0.18	5.55E-13
Education	Income env.-dep.?	-0.26	0.00E+00
Age	Born in village?	-0.05	2.65E-02
Sex	Born in village?	0.33	0.00E+00
Education	Born in village?	0.09	1.00E-04
Income env.-dep.?	Born in village?	0.01	5.99E-01
Age	Heard of climate change?	-0.09	4.43E-04
Sex	Heard of climate change?	0.11	5.69E-06
Education	Heard of climate change?	0.34	0.00E+00
Income env.-dep.?	Heard of climate change?	-0.15	3.43E-09
Born in village?	Heard of climate change?	0.07	5.68E-03

Table S6 Linear regression models of determinants of the magnitude of respondents' error in perceiving erosion for the year 2020. Model 1 includes socio-demographics (sex, age, education); model 2 includes village fixed effects

	Dependent variable:	
	Error 2020 (m)	
	(1)	(2)
Eros. extent 2020 (m)	0.74	0.82
	(0.49)	(0.78)
Eros. impact 2020 (n/y)	227.23***	152.66***
	(62.99)	(48.06)
Dist. from river (m)	1.01*	1.22***
	(0.56)	(0.33)
Income env.-dep.? (n/y)	171.19***	64.61
	(58.72)	(52.45)
Born in village? (n/y)	-66.40	-14.25
	(51.77)	(51.65)
Sex (f/m)	-157.78*	-131.48*
	(88.49)	(78.56)
Age: 31-40 yr	-28.03	-58.41
	(92.80)	(79.76)
Age: 41-50 yr	3.51	-21.55
	(106.64)	(83.29)
Age: 51-60 yr	-80.86	-87.68
	(88.76)	(86.47)
Age: 61+ yr	15.07	-12.15
	(106.85)	(88.55)
Educ: primary	-104.47	-87.34
	(66.57)	(68.18)
Educ: secondary	52.67	27.64
	(89.77)	(76.20)
Educ: SSC passed	-13.43	-110.43
	(144.07)	(131.34)
Educ: HSC passed	-151.49*	-163.60

	(84.60)	(123.80)
Educ: university	-136.79	-183.87
	(96.99)	(132.68)
Village 5		580.29***
		(190.22)
Village 6		-435.55**
		(171.52)
Village 7		-48.52
		(166.36)
Village 8		145.62
		(228.19)
Village 9		-116.78
		(176.93)
Village 10		68.68
		(282.99)
Village 11		64.79
		(165.58)
Village 12		44.36
		(180.89)
Village 13		29.15
		(180.87)
Village 14		155.79
		(174.61)
Village 15		-179.62
		(228.87)
Village 16		545.84**
		(216.51)
Village 17		-498.88
		(463.00)
Village 18		-369.77
		(620.56)
Village 19		502.07***
		(174.09)
Village 20		749.03***
		(159.96)
Village 21		817.94**
		(387.78)

Village 22		756.41***
		(157.25)
Village 23		236.96
		(163.45)
Village 24		-180.52
		(158.19)
Village 25		325.95*
		(194.16)
Village 26		-7.23
		(179.39)
Village 27		273.44
		(196.23)
Village 28		173.85
		(166.00)
Village 29		428.45**
		(199.46)
Village 30		428.19**
		(166.73)
Village 31		-130.18
		(174.38)
Village 32		55.57
		(168.09)
Village 33		-51.22
		(178.33)
Village 34		-280.19
		(200.44)
Village 35		47.63
		(201.58)
Village 36		35.21
		(210.69)
Intercept	659.17***	547.85***
	(119.51)	(149.77)
Village FE?	No	Yes
Observations	1,348	1,348
R^2	0.05	0.17
Adjusted R^2	0.04	0.14
Residual Std. Error	871.65 (df = 1332)	826.24 (df = 1300)

F Statistic	4.69*** (df = 15; 1332)	5.55*** (df = 47; 1300)
Note:	*p<0.1; **p<0.05; ***p<0.01	
	Standard errors clustered by village. Baseline age: 18-30 yr. Baseline education: no education. Baseline village: village 4. (m) – (meters), (n/y) – (no/yes), (f/m) – (female/male).	

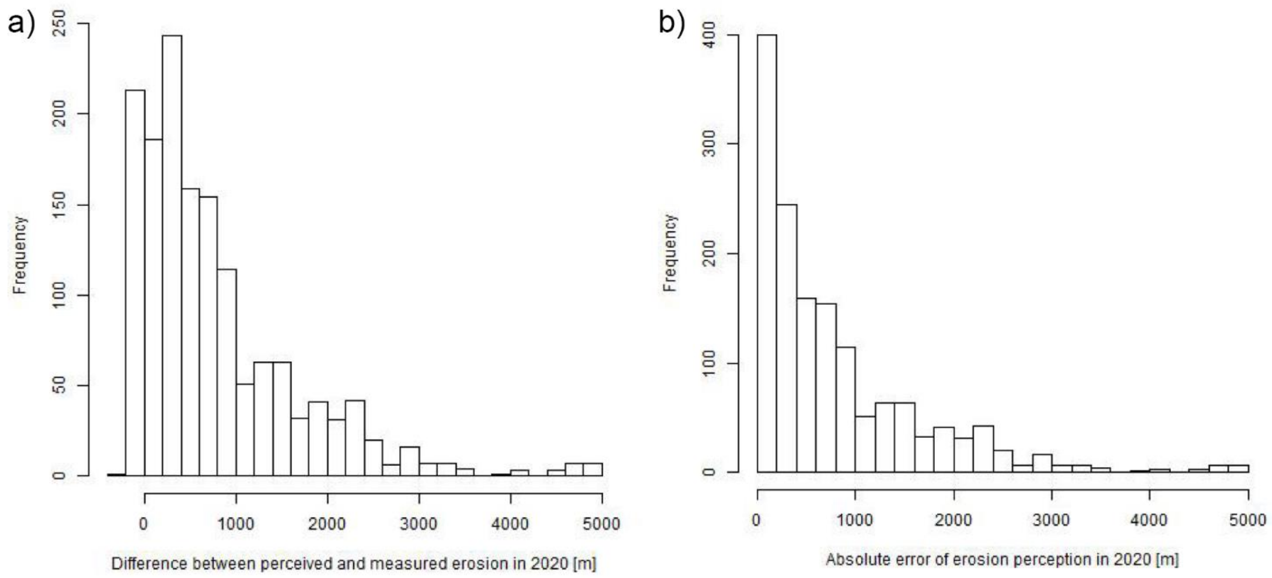

Fig. S5 Difference of perceived and measured erosion in a) actual and b) absolute values for 2020 (only values < 5000 m are plotted to enhance readability)

Table S7 Quantile regressions of the absolute error of the perception of the 2020 erosion extent, including socio-demographics (sex, age, education). Model numbers correspond to the deciles (e.g. model 1 corresponds to the 10th percentile, model 2 to the 20th percentile, etc.)

	Dependent variable:								
	Error (2020)								
	(1)	(2)	(3)	(4)	(5)	(6)	(7)	(8)	(9)
Eros. extent 2020 (m)	0.71***	0.95***	0.97***	0.94***	1.29***	1.44***	1.12***	0.29	-0.55
	(0.08)	(0.23)	(0.19)	(0.28)	(0.38)	(0.35)	(0.26)	(0.56)	(0.58)
Eros. impact 2020 (n/y)	22.78**	109.67***	135.82***	195.70***	219.35***	216.29***	328.50***	375.33***	378.84***
	(9.74)	(22.64)	(23.95)	(30.22)	(41.98)	(50.24)	(62.45)	(99.75)	(135.15)
Dist. from river (m)	0.19**	0.59***	0.75***	0.92***	1.29***	1.28***	1.58***	1.90***	2.20***
	(0.09)	(0.18)	(0.20)	(0.25)	(0.30)	(0.35)	(0.44)	(0.71)	(0.60)
Income env.-dep.? (n/y)	13.41*	53.84**	68.86***	117.22***	113.32**	168.96***	190.11***	289.61***	232.68**
	(7.28)	(22.03)	(25.93)	(31.54)	(45.33)	(54.86)	(64.39)	(104.90)	(99.20)
Born in village? (n/y)	-3.99	-22.92	-42.99	-65.19**	-74.45	-149.22**	-184.15***	-251.76**	-185.89*
	(8.54)	(25.70)	(26.98)	(33.04)	(47.83)	(59.98)	(59.04)	(107.02)	(99.88)
Sex (f/m)	-1.03	-17.11	-44.08	-68.36	-112.78	-72.29	-324.30	-420.21***	-262.28**
	(14.44)	(35.27)	(48.39)	(44.20)	(77.39)	(145.52)	(204.75)	(157.15)	(108.08)
Age: 31-40 yr	8.62	42.72	36.16	25.82	-23.25	-112.19	-66.28	-64.39	-127.14
	(11.04)	(27.81)	(42.39)	(54.10)	(83.46)	(92.65)	(117.20)	(151.90)	(174.73)
Age: 41-50 yr	2.90	59.66*	54.47	60.51	34.98	12.32	-19.61	-55.19	-23.52
	(12.10)	(31.72)	(42.31)	(63.00)	(95.01)	(101.04)	(108.25)	(163.68)	(194.48)
Age: 51-60 yr	-11.86	15.36	-5.11	-24.89	-68.46	-125.85	-82.74	-220.57	0.002
	(14.02)	(28.08)	(43.54)	(59.02)	(94.16)	(99.90)	(107.78)	(182.48)	(228.41)
Age: 61+ yr	1.42	29.00	-5.12	-8.43	-37.50	-102.12	-88.59	-33.76	11.38
	(12.88)	(25.64)	(42.87)	(59.98)	(97.40)	(99.42)	(116.52)	(195.32)	(204.57)

Educ: primary	4.77	-8.55	-13.51	-31.17	-101.96*	-117.24	-187.33**	-244.71*	-178.75
	(10.45)	(23.65)	(33.44)	(35.78)	(54.80)	(78.74)	(74.62)	(127.47)	(189.10)
Educ: secondary	4.51	15.66	0.21	15.00	-24.75	-77.63	-169.36	114.61	390.04*
	(10.03)	(35.39)	(38.31)	(51.29)	(57.91)	(59.37)	(113.56)	(233.63)	(212.71)
Educ: SSC passed	-15.75	-23.19	-63.66	-56.01	-152.78	33.62	-143.99	-48.13	-418.54***
	(11.73)	(42.54)	(45.38)	(41.97)	(155.64)	(145.57)	(97.72)	(174.49)	(141.96)
Educ: HSC passed	-8.30	-20.80	-66.35	19.26	-76.90	-103.64	-250.00	-153.57	-337.47**
	(19.37)	(51.94)	(63.18)	(86.49)	(68.20)	(89.93)	(190.68)	(212.12)	(131.48)
Educ: university	7.60	-37.69	-103.71**	-143.78**	-227.98*	-14.27	-105.26	210.80	45.19
	(35.55)	(31.69)	(40.94)	(58.26)	(126.49)	(208.22)	(88.24)	(362.79)	(264.70)
Intercept	-18.74	-25.83	78.03	156.00**	334.80***	544.89***	959.02***	1,365.78***	1,765.40***
	(18.71)	(35.12)	(58.42)	(71.22)	(111.30)	(165.55)	(222.16)	(202.32)	(227.37)
Observations	1,348	1,348	1,348	1,348	1,348	1,348	1,348	1,348	1,348

Note: *p<0.1; **p<0.05; ***p<0.01

Baseline age: 18-30 yr. Baseline education: no education. (m) – (meters), (n/y) – (no/yes), (f/m) – (female/male).

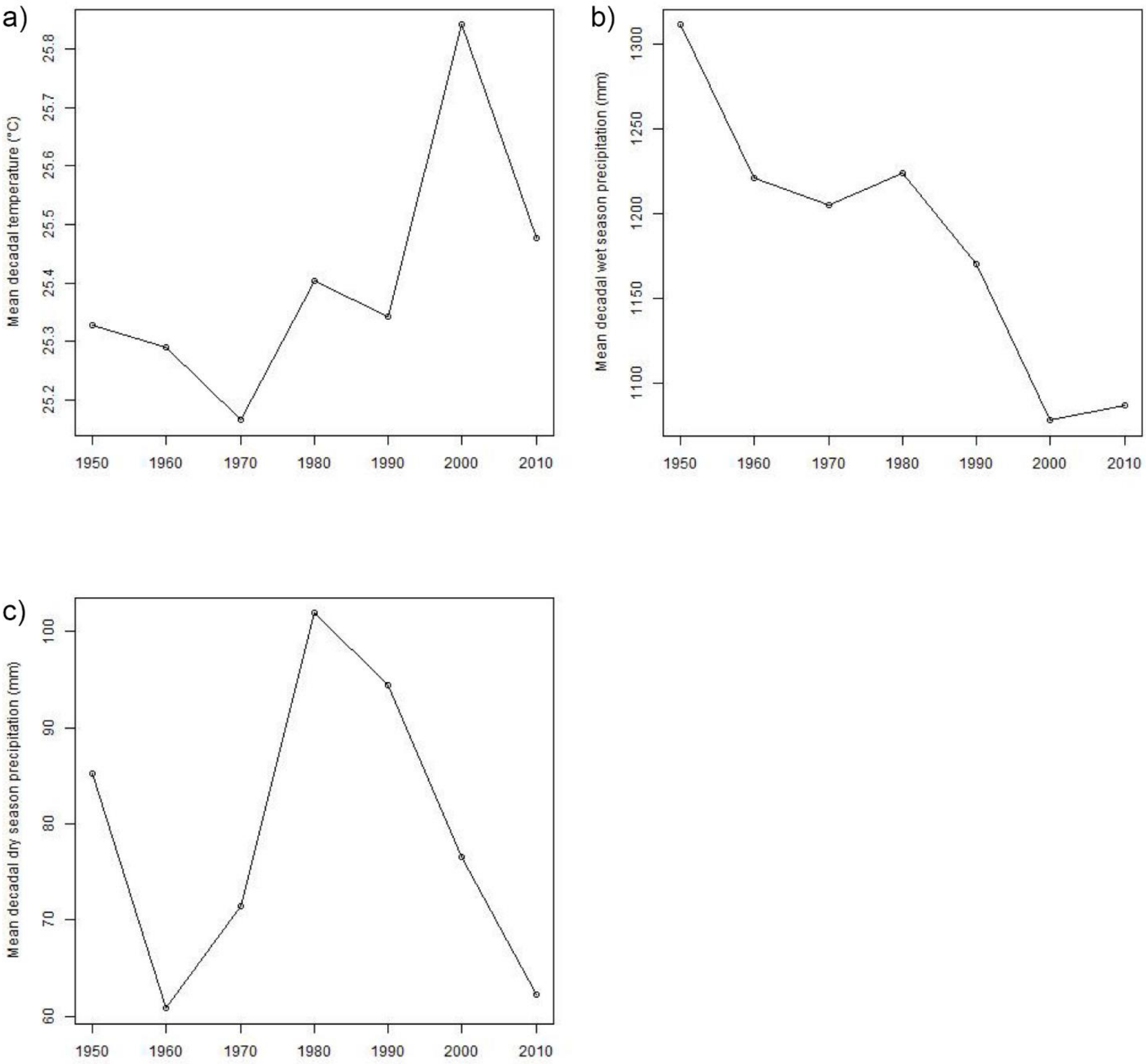

Fig. S6 Decadal mean of a) temperature, b) wet season precipitation and c) dry season precipitation in the study region. All points refer to the following decade (e.g., the first point refers to the decade 1950-1960)

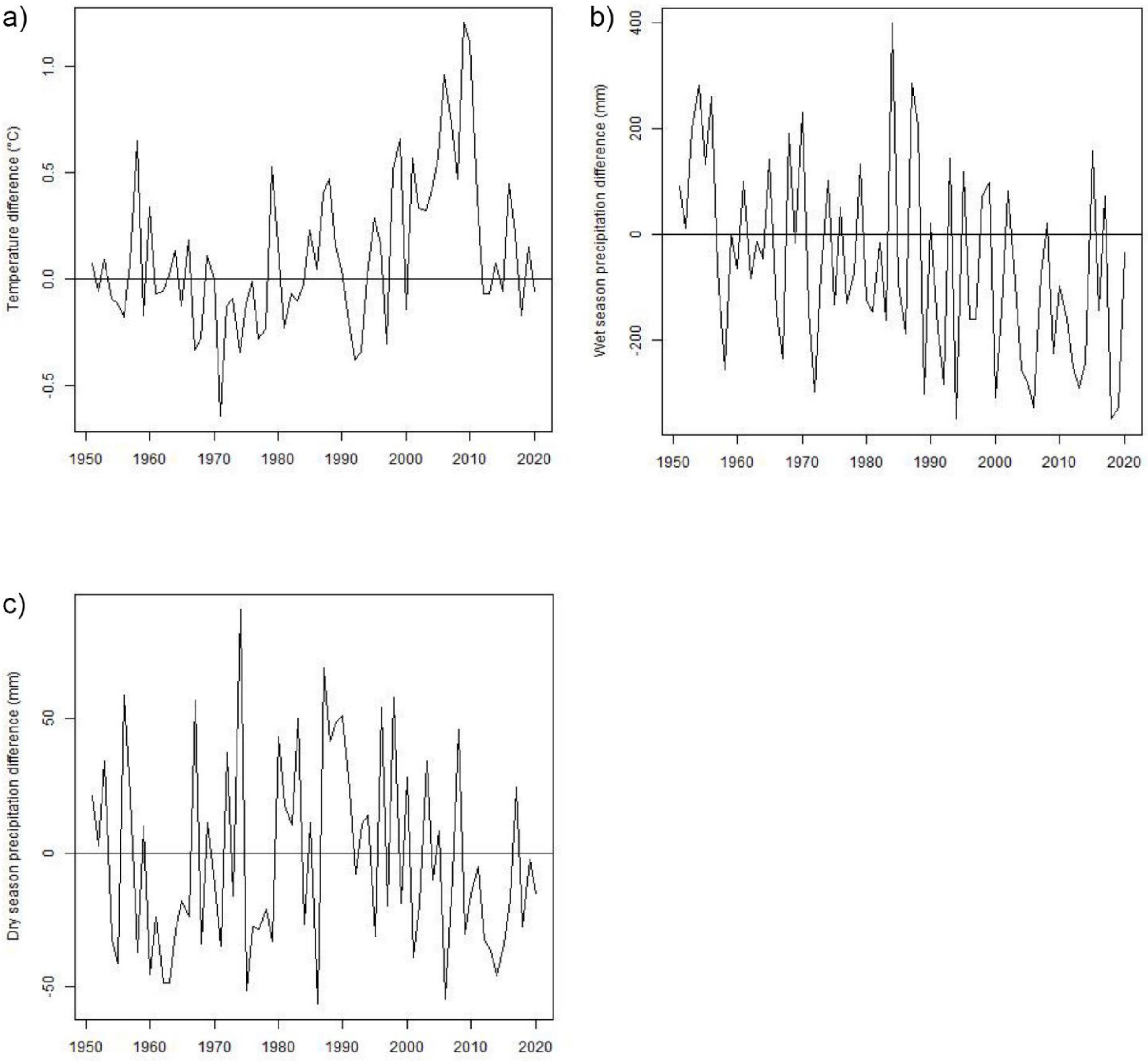

Fig. S7 a) Deviation of the mean yearly temperature from the mean temperature between 1951 and 1990. b) Deviation of the yearly wet season precipitation from the mean yearly wet season precipitation between 1951 and 1990. c) Deviation of the yearly dry season precipitation from the mean yearly dry season precipitation between 1951 and 1990

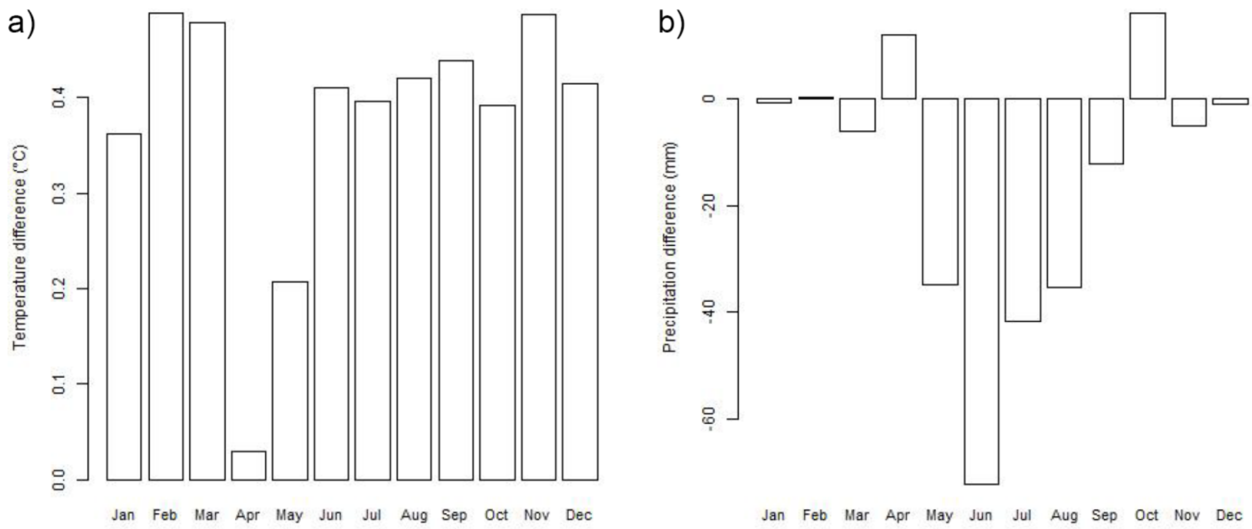

Fig. S8 Deviation of the mean monthly a) temperature and b) precipitation between 2001 and 2020 from the mean monthly temperature/precipitation between 1951 and 1990

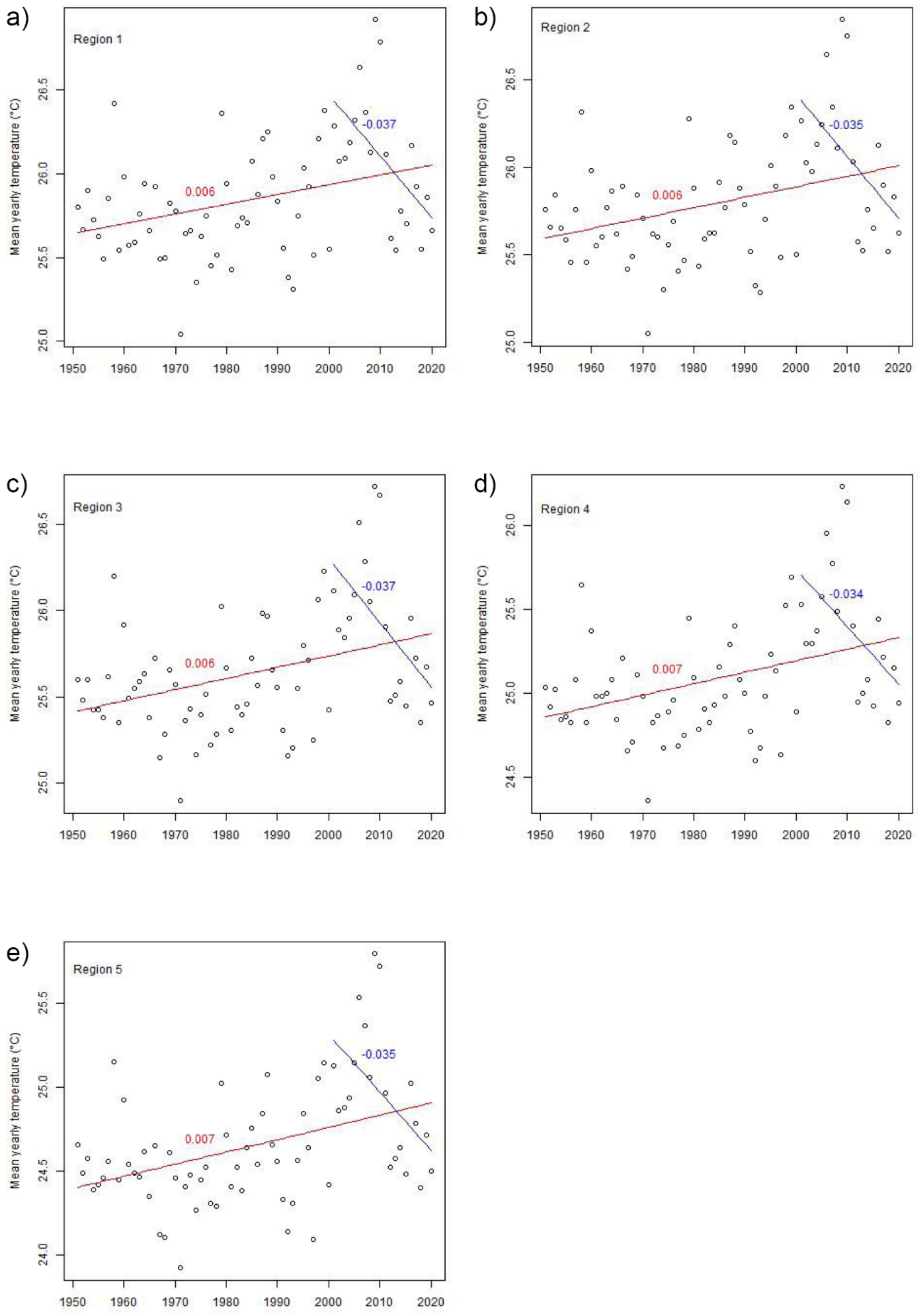

Fig. S9 Mean yearly temperature between 1950 and 2020 in the five CRU regions which cover the 36 study villages, including trend lines for the whole period (red) and for the past 20 years (blue line)

Table S8 Summary statistics of the temperature in the five CRU regions as well as for the average across all regions

**Variable**	**N**	**Mean**	**Std. Dev.**	**Min**	**Pctl. 25**	**Pctl. 75**	**Max**
Region 1 (south)	70	25.85	0.35	25.04	25.6	26.08	26.92
Region 2	70	25.8	0.35	25.05	25.56	26	26.84
Region 3	70	25.64	0.36	24.9	25.41	25.88	26.72
Region 4	70	25.1	0.36	24.36	24.85	25.3	26.23
Region 5 (north)	70	24.65	0.36	23.93	24.43	24.85	25.79
Average across regions	70	25.41	0.35	24.66	25.18	25.63	26.5

Table S9 Summary of the regression estimates of temperature trend lines for the periods 1950-2020 and 2001-2020 in the five CRU regions as well as for the average across all regions

	**Estimate (1951-2020)**	**p-value (1951-2020)**	**Estimate (2001-2020)**	**p-value (2001-2020)**
Region 1 (south)	0.006	0.004	-0.037	0.012
Region 2	0.006	0.003	-0.035	0.016
Region 3	0.006	0.002	-0.037	0.013
Region 4	0.007	0.001	-0.034	0.024
Region 5 (north)	0.007	0	-0.035	0.023
Average across regions	0.006	0.001	-0.036	0.017

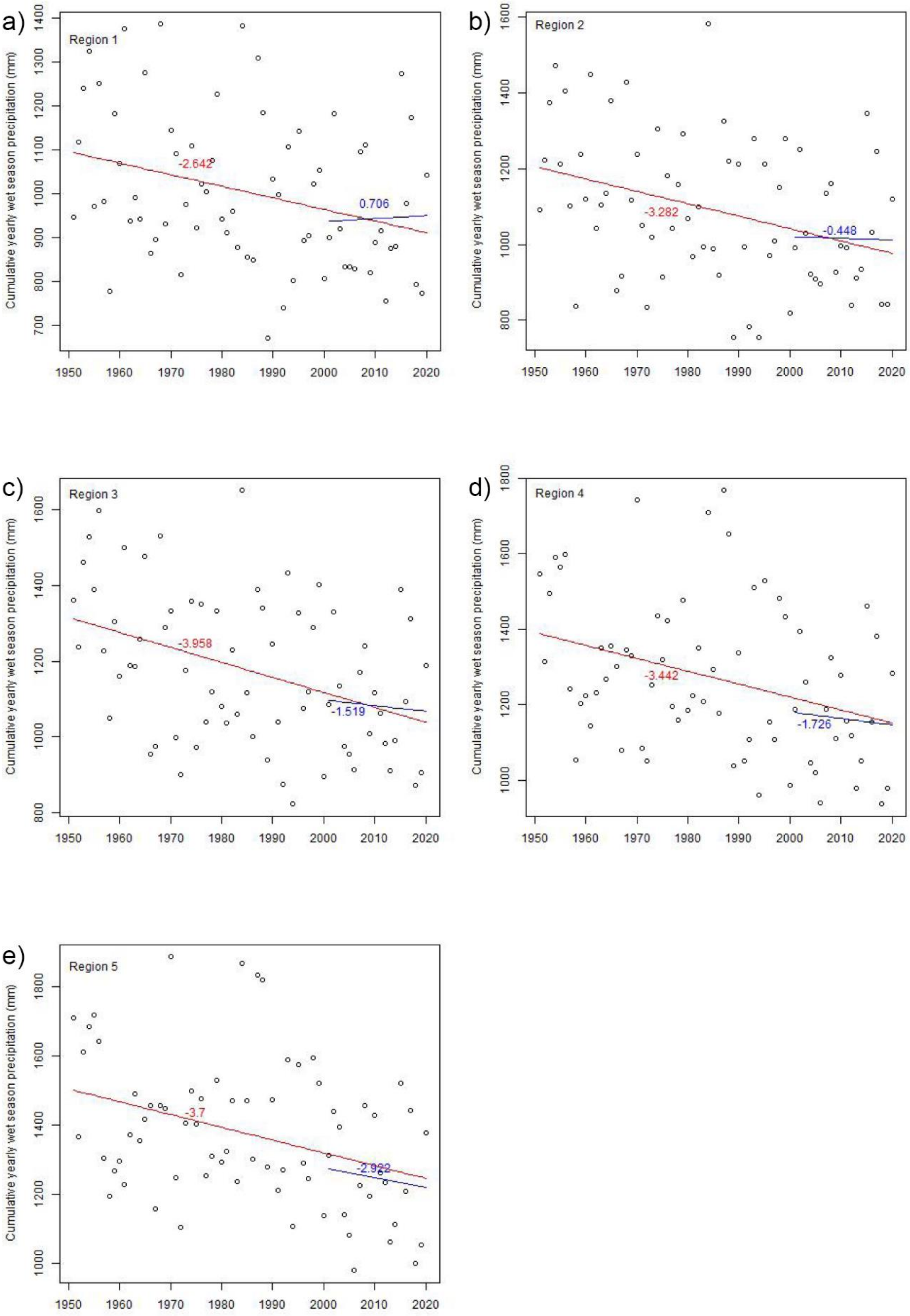

Fig. S10 Yearly wet season precipitation between 1950 and 2020 in the five CRU regions into which the 36 study villages fall, including trend lines for the whole period (red) and for the past 20 years (blue line)

Table S10 Summary statistics of wet season precipitation in the five CRU regions as well as for the average across all regions

**Variable**	**N**	**Mean**	**Std. Dev.**	**Min**	**Pctl. 25**	**Pctl. 75**	**Max**
Region 1 (south)	70	1001.95	171.75	670.1	877.45	1109.88	1385.6
Region 2	70	1089.77	191.03	754.1	929.33	1222.53	1582.2
Region 3	70	1175.27	199.51	823.4	1003.08	1330.98	1650.4
Region 4	70	1269.65	203.15	936.2	1112.73	1390.33	1767.8
Region 5 (north)	70	1373.03	207.57	979.2	1235.08	1474.6	1886.5
Average across regions	70	1181.93	180.25	888.92	1062.49	1327.75	1638.34

Table S11 Summary of the regression estimates of wet season precipitation trend lines for the periods 1950-2020 and 2001-2020, in the five CRU regions as well as for the average across all regions

	**Estimate (1951-2020)**	**p-value (1951-2020)**	**Estimate (2001-2020)**	**p-value (2001-2020)**
Region 1 (south)	-2.642	0.008	0.706	0.908
Region 2	-3.282	0.003	-0.448	0.94
Region 3	-3.958	0.001	-1.519	0.805
Region 4	-3.442	0.003	-1.726	0.787
Region 5 (north)	-3.7	0.002	-2.922	0.666
Average across regions	-3.405	0.001	-1.182	0.845

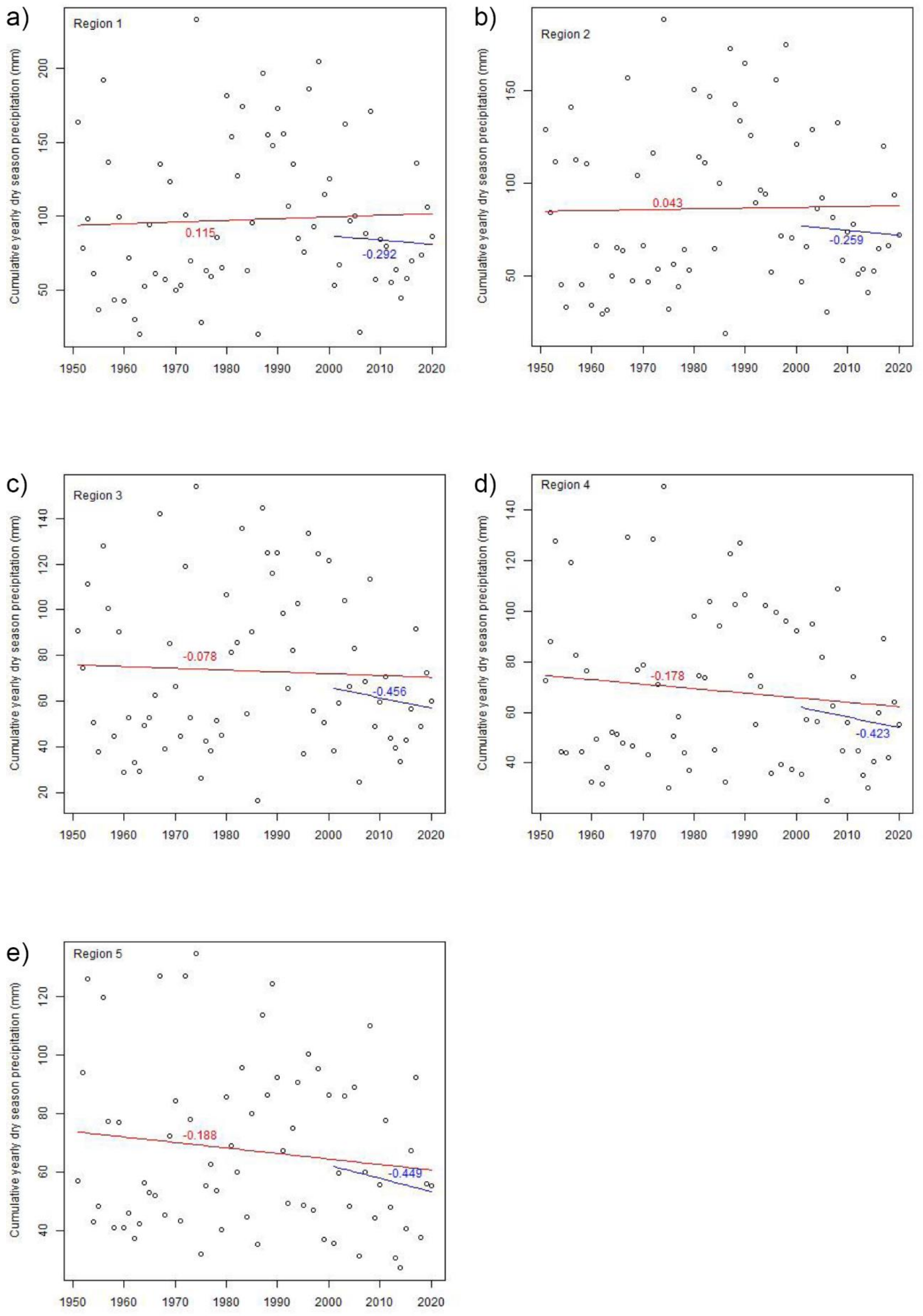

Fig. S11 Yearly dry season precipitation between 1950 and 2020 in the five CRU regions into which the 36 study villages fall, including trend lines for the whole period (red) and for the past 20 years (blue line)

Table S12 Summary statistics of dry season precipitation in the five CRU regions as well as for the average across all regions

Variable	N	Mean	Std. Dev.	Min	Pctl. 25	Pctl. 75	Max
Region 1 (south)	70	97.94	50.73	20.4	59.7	135.45	232.5
Region 2	70	86.24	41.9	18.8	52.55	115.65	187.9
Region 3	70	73.23	34.84	16.5	44.9	100.13	153.8
Region 4	70	68.37	30.4	25.1	44.15	91.3	149
Region 5 (north)	70	67.29	27.92	27.3	44.8	86.38	134.7
Average across regions	70	78.62	35.47	24.7	50.13	103.89	171.58

Table S13 Summary of the regression estimates of dry season precipitation trend lines for the periods 1950-2020 and 2001-2020, in the five CRU regions as well as for the average across all regions

	Estimate (1951-2020)	p-value (1951-2020)	Estimate (2001-2020)	p-value (2001-2020)
Region 1 (south)	0.115	0.705	-0.292	0.848
Region 2	0.043	0.863	-0.259	0.819
Region 3	-0.078	0.706	-0.456	0.627
Region 4	-0.178	0.325	-0.423	0.639
Region 5 (north)	-0.188	0.257	-0.449	0.629
Average across regions	-0.057	0.787	-0.376	0.723

Table S14 Determinants of respondents' perception of temperature, wet and dry season precipitation changes (linear regressions), including socio-demographic variables (sex, age, education). Models 2, 4 and 6 contain village fixed effects

	Dependent variable:					
	Temperature		Wet season precip.		Dry season precip.	
	(1)	(2)	(3)	(4)	(5)	(6)
Income env.-dep.? (n/y)	-0.18***	-0.12***	0.13**	0.05	-0.07	0.02
	(0.04)	(0.04)	(0.07)	(0.07)	(0.06)	(0.07)
Born in village? (n/y)	-0.01	0.001	-0.0001	0.01	0.02	0.04
	(0.04)	(0.04)	(0.06)	(0.07)	(0.06)	(0.06)
Heard of climate change? (n/y)	0.13**	0.11***	-0.13**	-0.04	-0.01	-0.05
	(0.05)	(0.04)	(0.07)	(0.07)	(0.06)	(0.07)
Sex (f/m)	-0.08	-0.06	-0.09	-0.08	0.11	0.11
	(0.07)	(0.06)	(0.10)	(0.10)	(0.10)	(0.10)
Age: 31-40 yr	-0.08	-0.08	-0.09	-0.10	-0.12	-0.09
	(0.05)	(0.06)	(0.10)	(0.10)	(0.10)	(0.10)
Age: 41-50 yr	-0.05	-0.06	-0.18	-0.22**	-0.14	-0.14
	(0.05)	(0.06)	(0.11)	(0.11)	(0.11)	(0.11)
Age: 51-60 yr	-0.02	-0.05	-0.27**	-0.29***	-0.10	-0.12
	(0.06)	(0.06)	(0.11)	(0.11)	(0.11)	(0.11)
Age: 61+ yr	-0.02	-0.05	-0.32***	-0.33***	-0.22**	-0.26**
	(0.06)	(0.07)	(0.12)	(0.11)	(0.11)	(0.11)
Educ: primary	-0.02	-0.05	-0.10	-0.14	-0.18**	-0.16*
	(0.04)	(0.05)	(0.09)	(0.09)	(0.08)	(0.09)
Educ: secondary	0.10	0.09	0.12	0.03	-0.12	-0.04
	(0.06)	(0.06)	(0.10)	(0.10)	(0.10)	(0.10)
Educ: SSC passed	-0.05	-0.04	-0.08	-0.10	-0.23	-0.17
	(0.13)	(0.10)	(0.17)	(0.17)	(0.16)	(0.16)
Educ: HSC passed	-0.19	-0.13	0.08	-0.02	-0.05	-0.04
	(0.12)	(0.09)	(0.16)	(0.16)	(0.15)	(0.15)
Educ: university	-0.01	-0.06	0.13	0.05	-0.45***	-0.40**
	(0.09)	(0.09)	(0.17)	(0.16)	(0.16)	(0.16)
Village 2		-0.56		-0.21		-0.28
		(0.34)		(0.60)		(0.57)
Village 3		-0.46		-0.30		-1.18*
		(0.39)		(0.68)		(0.65)

Village 4	0.60**	-0.68	-0.34
	(0.24)	(0.43)	(0.39)
Village 5	0.57**	-0.90**	-0.79*
	(0.25)	(0.44)	(0.41)
Village 6	0.46*	-0.86**	-0.34
	(0.25)	(0.43)	(0.40)
Village 7	0.63***	-0.24	-0.28
	(0.24)	(0.42)	(0.39)
Village 8	0.72***	-0.32	-0.14
	(0.24)	(0.42)	(0.39)
Village 9	0.43*	-0.63	0.39
	(0.25)	(0.43)	(0.40)
Village 10	0.75***	0.52	-0.60
	(0.28)	(0.48)	(0.45)
Village 11	0.58**	-0.04	-0.23
	(0.25)	(0.43)	(0.39)
Village 12	0.37	-0.69	0.05
	(0.25)	(0.43)	(0.40)
Village 13	0.54**	0.04	-0.48
	(0.25)	(0.44)	(0.40)
Village 14	0.50**	-0.51	-0.31
	(0.25)	(0.43)	(0.40)
Village 15	0.39	-1.24***	-0.01
	(0.27)	(0.47)	(0.44)
Village 16	0.64**	0.19	-0.10
	(0.27)	(0.46)	(0.43)
Village 17	-0.48	0.71	1.28*
	(0.37)	(0.76)	(0.72)
Village 18	0.08	0.39	0.51
	(0.39)	(0.68)	(0.65)
Village 19	0.36	-0.54	-0.63
	(0.25)	(0.43)	(0.40)
Village 20	0.52**	0.16	-0.67*
	(0.24)	(0.42)	(0.38)
Village 21	0.68**	0.30	-0.40
	(0.31)	(0.53)	(0.50)
Village 22	0.32	-0.11	-0.51
	(0.24)	(0.42)	(0.38)

Village 23		0.33		-0.40		-0.52
		(0.24)		(0.42)		(0.39)
Village 24		0.27		0.27		-0.86**
		(0.24)		(0.42)		(0.39)
Village 25		0.29		-0.46		-0.15
		(0.25)		(0.44)		(0.40)
Village 26		0.31		-0.01		-0.43
		(0.25)		(0.43)		(0.40)
Village 27		0.33		-0.66		-0.16
		(0.26)		(0.45)		(0.41)
Village 28		0.37		-0.57		-0.30
		(0.25)		(0.43)		(0.39)
Village 29		0.35		-0.63		-0.30
		(0.25)		(0.44)		(0.41)
Village 30		0.13		-0.49		-0.12
		(0.25)		(0.43)		(0.39)
Village 31		-0.04		-0.17		-0.20
		(0.24)		(0.42)		(0.39)
Village 32		0.29		-0.23		-0.26
		(0.24)		(0.42)		(0.39)
Village 33		0.22		-0.40		-0.57
		(0.25)		(0.43)		(0.40)
Village 34		0.26		-0.58		-0.51
		(0.25)		(0.44)		(0.40)
Village 35		0.22		-0.51		-0.36
		(0.26)		(0.45)		(0.42)
Village 36		0.30		-0.69		-0.55
		(0.25)		(0.43)		(0.40)
Intercept	4.72***	4.32***	3.68***	4.06***	2.93***	3.23***
	(0.08)	(0.24)	(0.13)	(0.42)	(0.12)	(0.38)
Village FE?	No	Yes	No	Yes	No	Yes
Observations	1,456	1,456	1,436	1,436	1,418	1,418
R^2	0.04	0.12	0.02	0.11	0.01	0.07
Adjusted R^2	0.03	0.09	0.01	0.08	0.002	0.04
Residual Std. Error	0.66 (df = 1442)	0.64 (df = 1407)	1.14 (df = 1422)	1.10 (df = 1387)	1.09 (df = 1404)	1.07 (df = 1369)
F Statistic	4.46*** (df = 13; 1442)	4.13*** (df = 48; 1407)	1.96** (df = 13; 1422)	3.44*** (df = 48; 1387)	1.25 (df = 13; 1404)	2.20*** (df = 48; 1369)

Note:	$^{*}p<0.1$; $^{**}p<0.05$; $^{***}p<0.01$
	Standard errors clustered by village. Baseline age: 18-30 yr. Baseline education: no education. Baseline village: village 1. (n/y) – (no/yes), (f/m) – (female/male).

Appendix C: Robustness checks and additional analyses

Since erosion affects certain villages more strongly than others, it might be the case that also perceptions of erosion differ between villages. Indeed, the average perception error per village ranges from 84 to 1805 meters (Fig. S12). One potential explanation could be that in villages which are strongly affected by erosion, erosion is a much-discussed topic, leading to a general overestimation of the actual erosion extent. However, the correlation between village level measured erosion and erosion error is weak or non-existent (Fig. S12).

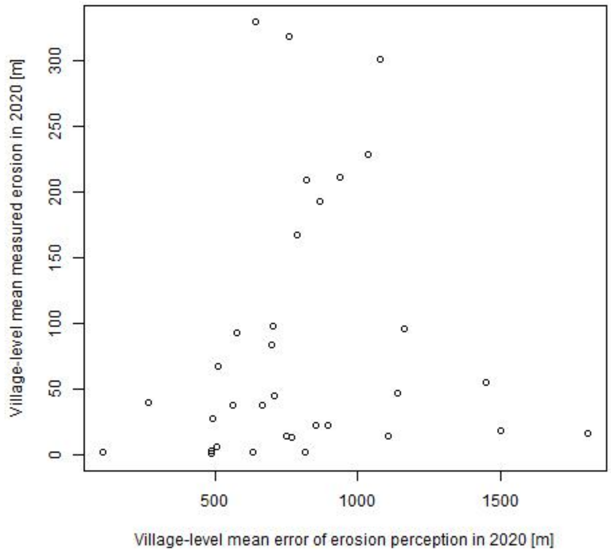

Fig. S12 Comparison of village level average measured erosion and average error of erosion perception for 2020

Irrespective of the underlying mechanism, it appears appropriate to account for any unobserved village-level heterogeneity by including village fixed effects into the models (see model 2 in Table S6). While most results remain substantively unchanged, the environmental dependence of the income does not exert a significant influence on the erosion error if village fixed effects are included. The effects of socio-demographics remain insignificant.

As a second robustness check, I log-transform the dependent error variable. This might improve the models given that the error variable spans a wide range of 0 to almost 5000 meters. Erosion impact retains a significant and substantive influence on the error magnitude, while in these models, also the objectively measured erosion extent increases the error significantly (Table S15). Distance from the river has a significant influence only in one model specification, while the income source and "born in the village" remain insignificant.

Table S15 Linear regression models of the logarithm of respondents' error in perceiving erosion for the year 2020. Model 2 contains village fixed effects. Full models including covariates (age, sex, education) are available upon request

	Dependent variable:	
	Log error 2020 (m)	
	(1)	(2)
Eros. extent 2020 (m)	0.003***	0.003**
	(0.001)	(0.001)
Eros. impact 2020 (n/y)	0.45***	0.32***
	(0.15)	(0.08)
Dist. from river (m)	0.001	0.001**
	(0.001)	(0.001)
Income env.-dep.? (n/y)	0.20	-0.03
	(0.12)	(0.08)
Born in village? (n/y)	-0.10	-0.12
	(0.09)	(0.08)
Intercept	1.72***	0.41*
	(0.34)	(0.23)
Village FE?	No	Yes
Observations	1,348	1,348
R^2	0.07	0.26
Adjusted R^2	0.06	0.23
Residual Std. Error	1.43 (df = 1332)	1.29 (df = 1300)
F Statistic	6.94*** (df = 15; 1332)	9.57*** (df = 47; 1300)
Note:	*p<0.1; **p<0.05; ***p<0.01	
	Standard errors clustered by village. (m) – (meters), (n/y) – (no/yes).	

Conducting an equivalent analysis as for the absolute error for a binary indicator of whether respondents perceived the five-year erosion trend correctly or not, I find a significant effect only for one parameter: Respondents for whom the measured erosion extent in 2020 was larger are more likely to perceive the trend correctly (Table S16).

Table S16 Logistic regression models of determinants of perceiving the five-year erosion trend wrongly. Model 2 includes socio-demographics (sex, age, education), model 3 includes village fixed effects

	Dependent variable:		
	Erosion change wrong (n/y)		
	(1)	(2)	(3)
Eros. extent 2020 (m)	-0.01***	-0.01***	-0.03***
	(0.004)	(0.001)	(0.004)
Eros. impact 2020 (n/y)	0.20	0.21*	0.24
	(0.15)	(0.12)	(0.17)
Dist. from river (m)	0.001	0.001	-0.001
	(0.001)	(0.001)	(0.001)
Income env.-dep.? (n/y)	-0.02	-0.11	-0.20
	(0.18)	(0.13)	(0.18)
Born in village? (n/y)	0.23	0.28**	0.38**
	(0.19)	(0.13)	(0.18)
Sex (f/m)		-0.12	-0.46*
		(0.20)	(0.27)
Age: 31-40 yr		-0.14	-0.21
		(0.21)	(0.28)
Age: 41-50 yr		-0.24	-0.24
		(0.21)	(0.29)
Age: 51-60 yr		-0.02	0.10
		(0.23)	(0.31)
Age: 61+ yr		0.06	0.30
		(0.23)	(0.32)
Educ: primary		-0.17	-0.06
		(0.17)	(0.23)
Educ: secondary		-0.44**	-0.31
		(0.19)	(0.25)
Educ: SSC passed		-1.16***	-0.98**
		(0.30)	(0.42)
Educ: HSC passed		-0.14	0.12

	(0.31)	(0.42)
Educ: university	-0.33	-0.46
	(0.34)	(0.48)
Village 2		-1.67
		(1.09)
Village 3		1.07*
		(0.64)
Village 4		0.78
		(0.48)
Village 5		0.15
		(0.47)
Village 6		0.56
		(0.49)
Village 7		5.69***
		(1.10)
Village 8		1.96**
		(0.85)
Village 9		0.46
		(0.45)
Village 10		7.88***
		(1.33)
Village 11		2.35***
		(0.50)
Village 12		3.88***
		(0.63)
Village 13		-0.12
		(0.48)
Village 14		-0.39
		(0.46)
Village 15		5.23***
		(1.10)
Village 16		1.88**
		(0.81)
Village 17		28.35
		(1,344.00)
Village 18		-3.29
		(1,947.76)

Village 19			1.94***
			(0.62)
Village 20			4.46***
			(0.64)
Village 21			4.57***
			(1.44)
Village 22			-1.18***
			(0.43)
Village 23			0.69*
			(0.40)
Village 24			2.23***
			(0.53)
Village 25			2.85***
			(0.79)
Village 26			2.46***
			(0.61)
Village 27			2.56***
			(0.80)
Village 28			1.64***
			(0.54)
Village 29			-0.66
			(0.83)
Village 30			1.99***
			(0.56)
Village 31			0.89*
			(0.49)
Village 32			4.50***
			(0.70)
Village 33			18.14
			(592.07)
Village 34			1.52**
			(0.70)
Village 35			0.58
			(0.56)
Village 36			-14.45
			(524.70)
Intercept	1.16**	1.52***	1.13**
	(0.46)	(0.27)	(0.50)

Village FE?	No	No	Yes
Observations	1,530	1,526	1,526
Log Likelihood	-859.79	-847.26	-519.90
Akaike Inf. Crit.	1,731.57	1,726.51	1,141.79

Note: *p<0.1; **p<0.05; ***p<0.01

Standard errors clustered by village. Baseline age: 18-30 yr. Baseline education: no education. Baseline village: village 1. (m) – (meters), (n/y) – (no/yes), (f/m) – (female/male).

Table S17 further characterizes the particularly inaccurate respondents by comparing respondents who overestimate the erosion extent by more than 1000 meters to respondents whose error is below 20 meters (binary operationalization). The actual erosion extent as well as having been personally affected significantly increase the likelihood of strongly overestimating the erosion extent. The other variables remain insignificant or inconsistent.

Table S17 Logistic regression models of determinants of overestimating the erosion extent by more than 1000 meters for the year 2020. Model 2 includes socio-demographic variables (sex, age, education). Model 3 contains village fixed effects

	Dependent variable:		
	Overestimator (n/y)		
	(1)	(2)	(3)
Eros. extent 2020 (m)	0.03***	0.03***	0.03***
	(0.01)	(0.01)	(0.01)
Eros. impact 2020 (n/y)	0.86***	0.87***	0.73**
	(0.28)	(0.25)	(0.33)
Dist. from river (m)	0.004	0.004**	0.004*
	(0.003)	(0.002)	(0.002)
Income env.-dep.? (n/y)	0.49	0.57**	0.05
	(0.34)	(0.27)	(0.36)
Born in village? (n/y)	-0.36	-0.23	-0.28
	(0.27)	(0.27)	(0.37)
Sex (f/m)		-0.53	-0.53
		(0.43)	(0.60)
Age: 31-40 yr		0.56	0.08
		(0.42)	(0.55)
Age: 41-50 yr		0.39	0.01
		(0.43)	(0.54)
Age: 51-60 yr		0.10	-0.13
		(0.45)	(0.58)
Age: 61+ yr		0.12	0.07
		(0.45)	(0.58)
Educ: primary		-0.18	-0.05
		(0.38)	(0.51)
Educ: secondary		0.13	0.21
		(0.43)	(0.56)
Educ: SSC passed		-0.69	-1.33
		(0.68)	(0.82)
Educ: HSC passed		-0.06	-0.28

	(0.69)	(0.95)
Educ: university	-0.26	-0.43
	(0.74)	(1.02)
Village 5		1.68
		(1.24)
Village 6		-19.14
		(2,287.81)
Village 7		0.17
		(0.84)
Village 8		13.64
		(2,171.37)
Village 9		-1.28
		(1.01)
Village 10		13.73
		(4,353.30)
Village 11		0.73
		(1.07)
Village 12		0.59
		(1.48)
Village 13		1.10
		(0.82)
Village 14		1.58
		(0.97)
Village 15		-1.68
		(1.73)
Village 16		1.18
		(1.00)
Village 17		4.77
		(10,754.01)
Village 18		2.87
		(7,406.29)
Village 19		1.69**
		(0.74)
Village 20		18.72
		(1,320.01)
Village 21		-7.35**
		(3.27)

Village 22			2.31**
			(0.94)
Village 23			17.96
			(2,112.76)
Village 24			-0.27
			(0.76)
Village 25			0.94
			(0.88)
Village 26			1.35
			(1.25)
Village 27			0.83
			(0.89)
Village 28			0.38
			(0.78)
Village 29			16.66
			(2,885.37)
Village 30			19.54
			(2,289.36)
Village 31			-0.46
			(0.84)
Village 32			0.28
			(0.96)
Village 33			18.30
			(4,016.39)
Village 34			0.53
			(0.88)
Village 35			0.25
			(1.36)
Village 36			14.51
			(2,619.79)
Intercept	-0.55	-0.45	-0.55
	(0.49)	(0.54)	(0.78)
Village FE?	No	No	Yes
Observations	491	491	491
Log Likelihood	-202.29	-199.17	-138.03
Akaike Inf. Crit.	416.58	430.34	372.06
Note:	*p<0.1; **p<0.05; ***p<0.01		

Standard errors clustered by village. Baseline age: 18-30 yr. Baseline education: no education. Baseline village: village 4. (m) – (meters), (n/y) – (no/yes), (f/m) – (female/male).

As a last robustness check, I estimate a model including place attachment. Place attachment might influence environmental perceptions if one sees it as a proxy for the emotional connection of people to their place of residence. Given that place attachment should be higher for people born in a village than for those who moved there at some point, the two variables “Born in village” and “Place attachment” can be understood as capturing the same underlying construct. Table S18 compares models including “Born in village” and “Place attachment” and shows that indeed, place attachment has a similar effect as “Born in village”: Higher levels of attachment are correlated to a lower perception error. The remaining effects remain substantively unchanged.

Table S18 Comparison of linear regression models of determinants of the magnitude of respondents' error in perceiving erosion for the year 2020 including "Born in village" (model 1) and "Place attachment" (model 2)

	Dependent variable:	
	Error 2020 (m)	
	(1)	(2)
Eros. extent 2020 (m)	0.74	0.76^{**}
	(0.49)	(0.32)
Eros. impact 2020 (n/y)	216.44^{***}	202.67^{***}
	(63.19)	(49.26)
Dist. from river (m)	0.98^{*}	0.99^{***}
	(0.54)	(0.31)
Income env.-dep.? (n/y)	167.63^{***}	167.72^{***}
	(52.84)	(48.69)
Born in village? (n/y)	-101.17^{*}	
	(53.88)	
Attachment (5 pt)		-63.86^{*}
		(32.97)
Intercept	510.63^{***}	737.46^{***}
	(81.65)	(157.16)
Village FE?	No	No
Observations	1,348	1,348
R^2	0.04	0.04
Adjusted R^2	0.04	0.04
Residual Std. Error (df = 1342)	872.00	872.22
F Statistic (df = 5; 1342)	11.83^{***}	11.69^{***}
Note:	*p<0.1; **p<0.05; ***p<0.01	
	Standard errors clustered by village. (m) – (meters), (n/y) – (no/yes).	

Appendix D: Pre-registration

D.1 Anonymized version of pre-analysis plan

Linking past and future. Comparing individual perceptions of environmental change with actual data

Individuals' perceptions of regional climate change reflect their judgments and awareness of global climate change and are responsible for shaping their adaptive responses (including migration). It is thus essential to consider these perceptions for the design of strategies to reduce vulnerability and increase resilience. In this part of the project, we examine the relationship between objective indicators and individuals' perceptions of both past occurrences and future likelihood of environmental change:

> *RQ1: How do individual perceptions of past environmental events compare with actual data?*
>
> *RQ2: How do individual perceptions of the risk of future environmental events compare with actual data?*

The literature seems to agree that perceptions about temperature are more consistent with meteorological evidence than perceptions about rainfall (de Longueville et al., 2020; Madhuri & Sharma, 2020; though see Marlon et al., 2019). Despite the wealth of studies comparing scientific assessments with individuals' perceptions of climate change, there is no consensus regarding this relationship in the literature. While some studies show that actual weather data and reported data agree (Kosmowski et al., 2016; Shrestha et al., 2019), they disagree in others (Meze-Hausken, 2004; Moyo et al., 2012; Sutcliffe et al., 2016). Moreover, a systemic comparison has not yet been conducted in the realm of climate events such as floods, storms or riverbank erosion (Howe et al., 2019).

Unlike rapid onset climate-related hazards such as floods and hurricanes, riverbank erosion also occurs in a gradual manner. Given its gradual occurrence, riverbank erosion resembles sea level rise and consequently, it could be perceived as both temporally and spatially distant by the people who reside along the river (Covi & Kain, 2016). A recent study by Shao et al. (2020) compares public expectations

and scientific estimations of sea level rise in five states of the US Gulf Coast region and finds that coastal residents in states that have experienced faster sea level rise in the past are more optimistic about future sea level rise, thereby underestimating its magnitude compared to those experiencing slower sea level rise. Consequently, the perception of gradual riverbank erosion as being distant in time and perhaps space could make it seem less relevant. This perception, however, would hinder timely adaptive actions at both individual and aggregate levels to cope with riverbank erosion.

To answer the first research question, we will consider five different environmental indicators: rainfall; monsoon intensity; temperature; floods; and riverbank erosion. Note that these comprise both gradual (temperature) and sudden-onset environmental changes (floods, sudden-onset erosion). Given the evidence presented above, we expect that respondents perceive only sudden-onset events correctly, but not gradual ones.

H1: Individuals do not perceive gradually occurring environmental changes accurately.

H2: Individuals perceive sudden-onset environmental changes accurately.

For the five environmental indicators, we will collect objective data on their development in the past years. In the household survey, we ask respondents about their perceptions of the occurrence and severity of erosion and floods in the last three years as well as the development of the five above-mentioned indicators over the past 20 years. This will give us a subjective measure for the five indicators, which we can compare to the objective data reporting these events.

We will assess the actual extent of erosion ourselves using radar satellite imagery. Likewise, we will assess the flood extent ourselves using radar satellite imagery, with a temporal resolution of the radar images of 6-12 days. Additional information on floods will be drawn from official government reports and maps on the extent, duration and severity of monsoon flooding (e.g., from the *Flood Forecasting and Warning Center*). Official maps can, however, only be used if they are detailed enough to give

accurate information on flood affectedness at a single GIS location. Precipitation data will be taken from the CRU time series data set.

To answer the second research question, we will consider two environmental indicators: floods and sudden-onset erosion. Similar to the first research question, we will compare objective and subjective indicators. We have no basis to formulate hypotheses about their perceptions of future environmental events, and hence proceed in an explorative fashion. In terms of erosion, we will ask respondents about their expectations of erosion occurring in their village in the next two monsoon seasons. We can compare these perceptions to the prediction of erosion risk provided by the Center for Environment and Geographic Information Services (CEGIS in Dhaka, Bangladesh). In terms of floods, we will follow the same procedure, but limit the exercise to one year ahead given that, to our knowledge, no predictions of flood risk exist two years ahead. We will use as an indicator the risk of being affected by a “moderate flood” and a “severe flood”, which we operationalize by the flood extent of a 10 and 50 year flood, respectively. We still need to verify whether such a prediction is also available for the Bangladesh Monsoon and whether it also comprises information on monsoon – and accordingly flood – intensity.

The respondents’ perceptions of future environmental change is likely to be affected by different factors, including the respondents’ spatial distance and familiarity with the event, the relevance for her or his livelihood, education levels as well as external risk communication (cf. section X).

In a last step, we will combine the results from both research questions to investigate whether respondents are able to use past experiences of environmental change to infer on potential occurrences in the future. Such evidence on links between past experiences and perceptions of future risk is highly relevant in times of changing climate.

D.2 Modifications to the pre-analysis plan

I partially adapted the pre-analysis plan and report the differences here for reasons of transparency.

- Scope: Initially, the paper was conceived to cover perceptions of both past changes as well as future risks. To keep the paper more focused, I only analyzed the above-mentioned RQ1. RQ2 (perceptions of future risk) will be covered in a separate paper project.
- Environmental indicators: Since reliable objective measured data on floods and monsoon intensity is not available at the level of analysis (households / villages), I could not compare the respondents' perceptions of these events to measured data.
- Theory: At the time of writing the pre-analysis plan, I was not yet aware of the two psychological theories applied to derive expectations on the accuracy of environmental perceptions (dual-process theory and construal-level theory). Hence, the hypotheses put forward in the theory section of this paper are opposite to those in the pre-analysis plan.

References

Alam, G. M. M., Alam, K., Mushtaq, S., & Clarke, M. L. (2017). Vulnerability to climatic change in riparian char and river-bank households in Bangladesh: Implication for policy, livelihoods and social development. *Ecological Indicators*, *72*, 23–32. https://doi.org/10.1016/j.ecolind.2016.06.045

CEGIS. (2018). *Update, improve and extend the erosion forecasting and warning tools in the three main rivers.* Center for Environment and Geographic Information Services.

Covi, M. P., & Kain, D. J. (2016). Sea-Level Rise Risk Communication: Public Understanding, Risk Perception, and Attitudes about Information. *Environmental Communication*, *10*(5), 612–633. https://doi.org/10.1080/17524032.2015.1056541

Crawford, T. W., Rahman, M. K., Miah, M. G., Islam, M. R., Paul, B. K., Curtis, S., & Islam, M. S. (2020). Coupled Adaptive Cycles of Shoreline Change and Households in Deltaic Bangladesh: Analysis of a 30-Year Shoreline Change Record and Recent Population Impacts. *Annals of the American Association of Geographers*, 1–23. https://doi.org/10.1080/24694452.2020.1799746

de Longueville, F., Ozer, P., Gemenne, F., Henry, S., Mertz, O., & Nielsen, J. Ø. (2020). Comparing climate change perceptions and meteorological data in rural West Africa to improve the understanding of household decisions to migrate. *Climatic Change.* Advance online publication. https://doi.org/10.1007/s10584-020-02704-7

Freihardt, J., & Frey, O. (2023). Assessing riverbank erosion in Bangladesh using time series of Sentinel-1 radar imagery in the Google Earth Engine. *Natural Hazards and Earth System Science*, *23*(2), 751–770. https://doi.org/10.5194/nhess-23-751-2023

Howe, P. D., Marlon, J. R., Mildenberger, M., & Shield, B. S. (2019). How will climate change shape climate opinion? *Environmental Research Letters*, *14*(11), 113001. https://doi.org/10.1088/1748-9326/ab466a

Islam, M. S [M. S.], Sultana, S., Saifunnahar, & Miah, M. A. (2015). Adaptation of Char Livelihood in Flood and River Erosion Areas through Indigenous Practice: A Study on Bhuapur Riverine Area in Tangail. *Journal of Environmental Science and Natural Resources*, *7*(1). https://doi.org/10.3329/jesnr.v7i1.22138

Kosmowski, F., Leblois, A., & Sultan, B. (2016). Perceptions of recent rainfall changes in Niger: a comparison between climate-sensitive and non-climate sensitive households. *Climatic Change*, *135*(2), 227–241. https://doi.org/10.1007/s10584-015-1562-4

Madhuri, & Sharma, U. (2020). How do farmers perceive climate change? A systematic review. *Climatic Change*, *162*(3), 991–1010. https://doi.org/10.1007/s10584-020-02814-2

Marlon, J. R., van der Linden, S., Howe, P. D., Leiserowitz, A., Woo, S. H. L., & Broad, K. (2019). Detecting local environmental change: the role of experience in shaping risk judgments about global warming. *Journal of Risk Research*, *22*(7), 936–950. https://doi.org/10.1080/13669877.2018.1430051

Meze-Hausken, E. (2004). Contrasting climate variability and meteorological drought with perceived drought and climate change in northern Ethiopia. *Climate Research*, *27*, 19–31. https://doi.org/10.3354/cr027019

Moyo, M., Mvumi, B. M., Kunzekweguta, M., Mazvimavi, K., Craufurd, P., & Dorward, P. (2012). Farmer perceptions on climate change and variability in semi-arid Zimbabwe in relation to climatology evidence. *African Crop Science Journal*, *20*, 317–335.

Sarker, M. H., Thorne, C. R., Aktar, M. N., & Ferdous, M. R. (2014). Morpho-dynamics of the Brahmaputra–Jamuna River, Bangladesh. *Geomorphology*, *215*, 45–59. https://doi.org/10.1016/j.geomorph.2013.07.025

Shao, W., Moftakhari, H., & Moradkhani, H. (2020). Comparing public perceptions of sea level rise with scientific projections across five states of the U.S. Gulf Coast region. *Climatic Change*, *163*(1), 317–335. https://doi.org/10.1007/s10584-020-02893-1

Shrestha, U. B., Shrestha, A. M., Aryal, S., Shrestha, S., Gautam, M. S., & Ojha, H. (2019). Climate change in Nepal: a comprehensive analysis of instrumental data and people’s perceptions. *Climatic Change*, *154*(3-4), 315–334. https://doi.org/10.1007/s10584-019-02418-5

Sutcliffe, C., Dougill, A. J., & Quinn, C. H. (2016). Evidence and perceptions of rainfall change in Malawi: Do maize cultivar choices enhance climate change adaptation in sub-Saharan Africa? *Regional Environmental Change, 16*(4), 1215–1224. https://doi.org/10.1007/s10113-015-0842-x
